# TIP60 promotes chemoresistance by limiting intracellular platinum accumulation and enhancing removal of cisplatin-DNA adducts

**DOI:** 10.1016/j.jbc.2026.113338

**Published:** 2026-07-16

**Authors:** Akshay Hira, Michael P. Craig, Caroline McLaughlin, Jin Zhang, John J. Turchi, Michael G. Kemp, Ramzi W. Nahhas, Madhavi P. Kadakia

**Affiliations:** 1 Department of Biochemistry and Molecular Biology, Wright State University Boonshoft School of Medicine, Dayton, Ohio, USA; 2 Department of Biochemistry, Molecular Biology and Pharmacology, Indiana University, Indiana School of Medicine, Indianapolis, Indiana, USA; 3 Department of Pharmacology and Toxicology, Wright State University Boonshoft School of Medicine, Dayton, Ohio, USA; 4 Department of Population and Public Health Sciences, Wright State University Boonshoft School of Medicine, Dayton, Ohio, USA; 5 Department of Psychiatry, Wright State University Boonshoft School of Medicine, Dayton, Ohio, USA

**Keywords:** TIP60, cisplatin-DNA adducts, ABCC1, chemoresistance, DNA damage response, XPC, SCC

## Abstract

Cisplatin resistance is a major barrier to effective treatment of squamous cell carcinoma (SCC) including cutaneous SCC and Head and neck SCC, where resistance develops in more than half of advanced cases. Our previous work demonstrated that genetic knockdown or pharmacological inhibition of TIP60 (KAT5), a histone acetyl transferase, sensitizes cisplatin-resistant SCC cells, induces cell cycle arrest and promotes cell death, suggesting a key role for TIP60 in mediating resistance. Here, we use cisplatin-sensitive and -resistant SCC cell lines, together with siRNA-mediated gene silencing, stable overexpression, pharmacological inhibition, immunodot-blot assays, and ICP-MS to demonstrate that TIP60 promotes resistance through two complementary pathways: (1) upregulation of the efflux transporter ABCC1, which reduces intracellular cisplatin accumulation, and (2) increased expression of XPC, a key component of the nucleotide excision repair pathway, involved in recognition and removal of cisplatin-DNA adducts. Elevated TIP60 levels correlate with reduced cisplatin-DNA adduct levels, enhanced removal of cisplatin-DNA adducts and increased cell survival in resistant lines. TIP60 depletion reduces ABCC1 expression and increases cisplatin-DNA adduct levels, effects similarly observed with the ABCC1 inhibitor, MK-571. In parallel, TIP60 knockdown impairs removal of cisplatin-DNA adducts and reduces expression of multiple DNA damage response (DDR) genes, including XPC. Combined inhibition of TIP60 with spironolactone (targeting XPB/NER) or with MK-571 further reduces cell survival and increases cell death in resistant cells. These findings establish TIP60 as a regulator of cisplatin resistance that integrates drug efflux and DNA repair pathways, highlighting TIP60 inhibition as a promising therapeutic strategy to overcome platinum resistance in SCC.

Cisplatin is a well-established chemotherapy agent known for its ability to induce DNA damage *via* the formation of platinum-DNA adducts (1). Despite its clinical success, the long-term effectiveness of cisplatin is often limited, particularly in advanced and non-resectable cutaneous squamous cell carcinoma (cSCC), where over half of the cases develop resistance (2, 3, 4). Similarly, prolonged and recurrent use of cisplatin in heterogeneous head and neck squamous cell carcinoma (HNSCC) often leads to the emergence of resistant tumor subpopulations and therapeutic failure (1, 2, 3). The varied mechanisms driving acquired cisplatin resistance include the upregulation of oncogenic drivers, reduced intracellular drug levels resulting from decreased uptake or increased efflux, increased DNA damage repair or increased drug detoxification by glutathione and metalloproteins (5). A deeper understanding of these complex resistance mechanisms is essential for developing effective, personalized treatments for SCC.

TIP60 (KAT5) is a highly conserved histone acetyltransferase and the catalytic core of the NuA4 chromatin remodeling complex (6). It plays a central role in maintaining genomic stability by acetylating histones and non-histone proteins, relaxing chromatin and facilitating both homologous recombination and non-homologous end joining during DNA repair (7, 8). TIP60 also acts as a transcriptional co-activator for factors such as p53, NF-κB, and Myc, regulating the expression of DNA repair and stress-response genes (9). Further, we previously showed that TIP60 acetylates and stabilizes ΔNp63α, an oncogenic transcription factor reported to regulate several genes in DNA damage response (DDR) signaling (10, 11). Beyond DNA repair, TIP60 influences cell-cycle progression, apoptosis, stem cell maintenance, and metabolic regulation, integrating multiple signaling pathways that determine cell fate (12). Its dysregulation has been linked to cancer progression and chemoresistance (13), making TIP60 an attractive target for therapeutic intervention. Together, these functions suggest that TIP60 serves as a hub for coordinating multiple processes relevant to the cisplatin response.

A well-characterized chemoresistance mechanism involves the imbalance between drug importers and exporters that regulate intracellular cisplatin levels (14). Among the ATP-binding cassette (ABC) transporters family, several exporters including MRP1 (ABCC1), MRP2, MRP3, and MRP5 ABC exporters are frequently upregulated in cisplatin resistant cancer cells (15, 16, 17, 18, 19). Notably, ABCC1 is overexpressed in multiple drug-resistant cancer cell lines and tumor types including leukemia, breast, prostate and lung cancers where its expression is correlated with poor response to chemotherapy (20, 21). In HNSCC, ABCC1 functions as a cisplatin exporter and its expression is strongly associated with chemoresistance (22, 23). Previous studies have shown that ΔNp63α upregulates ABCC1 transcript levels in HNSCC (22, 24), while TIP60 has been reported to increase expression of another exporter, P-glycoprotein (MDR1) (25). These findings suggest a potential role for the TIP60/ΔNp63α axis in promoting cisplatin resistance by increasing drug efflux.

In addition to drug export, cisplatin treatment activates multiple DDR pathways due to formation of intra- and inter-strand cisplatin-DNA crosslinks (5). Numerous DDR-related genes are differentially regulated in cisplatin-resistant cells (26, 27). Interestingly, both ΔNp63α and TIP60 have independently been implicated in DDR regulation and cisplatin resistance (8, 28). ΔNp63α transcriptionally regulates key DDR players such as FANCD2 and RAD18 (29), while TIP60 upregulates FANCD2 and BRCA1 expression (13). TIP60 is recruited to sites of DNA damage where it acetylates ATM, initiating ATM-dependent signaling contributing to replication fork stability (8, 30).

Bulky DNA lesions in the form of cisplatin-DNA intrastrand adducts are primarily repaired through the nucleotide excision repair (NER) pathway (14, 31, 32). Both ΔNp63α and TIP60 have been linked to NER regulation. p63 has been shown to regulate the expression of XPC, a pivotal component of the NER damage recognition complex in response to UV-induced damage (33, 34). Similarly, TIP60 has been implicated in NER-mediated repair of UV-induced lesions (35). These collective findings suggest a previously unexplored crosstalk between ΔNp63α and TIP60 in modulating NER-dependent DNA repair pathways underlying cisplatin resistance.

We previously identified TIP60 as an upstream regulator of ΔNp63α and showed that high levels of both proteins correlate with cisplatin resistance in SCC cell lines (10, 11). Moreover, TIP60 depletion or pharmacologic inhibition decreases ΔNp63α acetylation, induces cell cycle arrest, and promotes apoptotic cell death in cisplatin-resistant cells (10). In the current study, we demonstrate, for the first time, that pharmacological inhibition of TIP60 sensitizes cisplatin-resistant SCC cells by impairing cisplatin-DNA adduct repair, reducing intracellular cisplatin levels and decreasing cell survival. Taken together, these findings establish TIP60 as a key mediator of cisplatin resistance and a promising therapeutic target for treating drug-resistant cutaneous SCC and Head and Neck SCC.

## Results

### Cisplatin-resistant cells exhibit reduced levels of cisplatin-DNA adducts compared to parental cells

**Figure 1:**
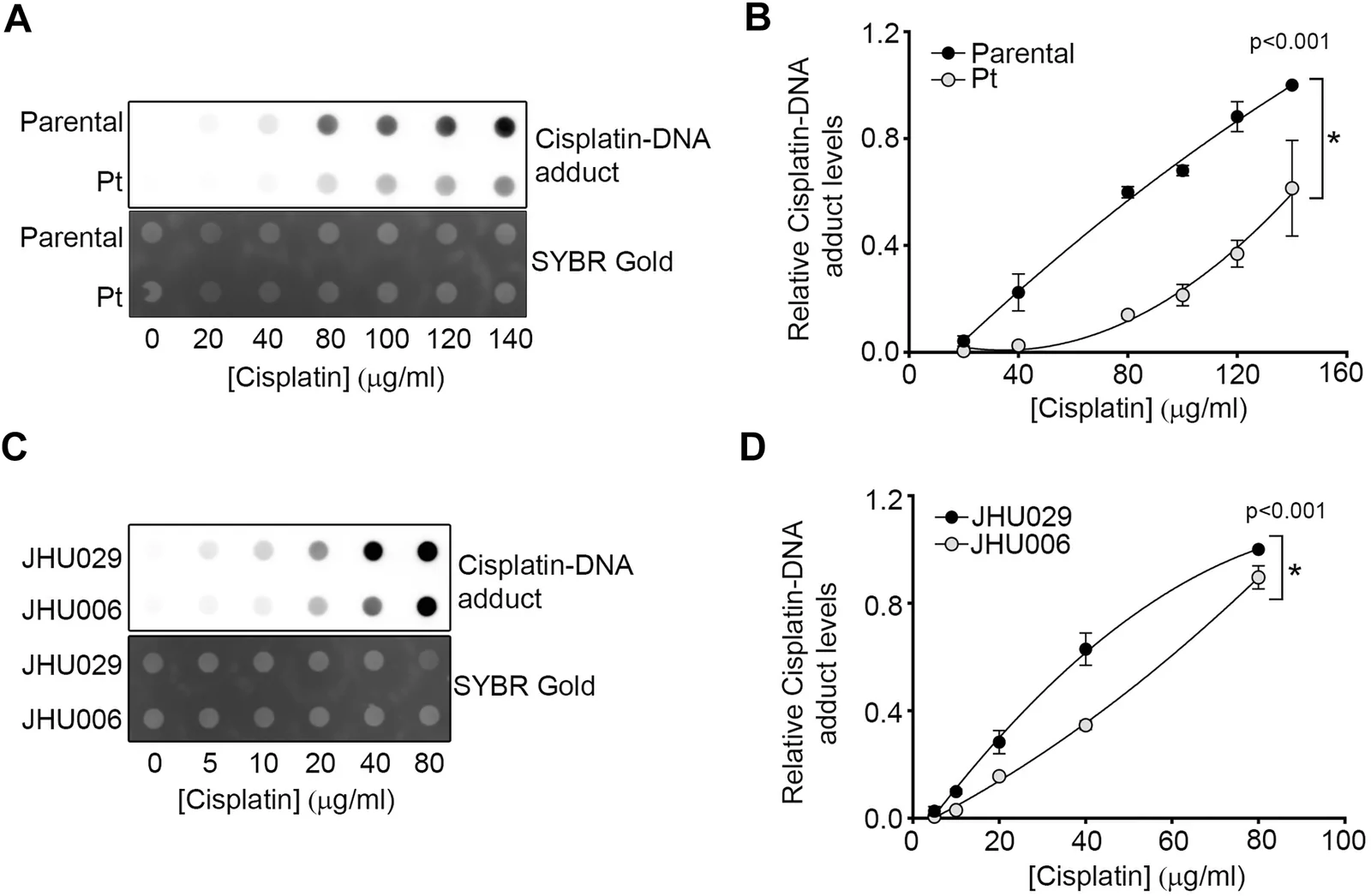
**Cisplatin resistant cells exhibit reduced 1,2-d(GpG) cisplatin-DNA levels.***A, C*, A431 Parental and Pt cells (*A*) were pulsed with 0, 20, 40, 80, 100, 120 and 140 μg/ml cisplatin (corresponding to 0, 66, 133, 266, 332, 399, 465 μΜ respectively), and JHU029 and JHU006 cells (*C*) were pulsed with 0, 5, 10, 20, 40 and 80 μg/ml cisplatin (corresponding to 0, 17, 33, 66, 133 and 266 μΜ, respectively) for 2 h. Following treatment, cells were washed with PBS and incubated in cisplatin free complete medium for 48 h after which genomic DNA was isolated. Immuno-dot blots of genomic DNA were probed with an antibody that specifically recognizes 1,2-d(GpG) cisplatin-DNA adducts and SYBR Gold staining was used to confirm equal DNA loading. *B, D*, densitometric quantitation of cisplatin adduct levels relative to peak adduct conditions (140 μg/ml (465 μM) in *A*, 80 μg/ml (266 μM) in *C*). The measured signal represents cisplatin-DNA adduct levels following pulse exposure, reflecting combined effects of uptake, efflux, and DNA reactivity during the treatment window. Regression lines shown are third order polynomial (cubic) lines of best fit. Error bars indicate ±1 S E M from three biological replicates. Dose–response curves were compared using a quadratic mixed-effects model with dose-dependent variance. ∗*p* ≤ 0.05 between the fitted dose response curves for each cell line.

We previously demonstrated that TIP60 levels are elevated in cisplatin resistant cells compared to cisplatin sensitive cells (10). Cisplatin resistance is multifactorial arising from either reduced drug uptake, enhanced nucleotide excision repair, altered apoptotic signaling, or increased tolerance to DNA damage and these mechanisms can vary across cell lines (1, 5, 36, 37). To determine if resistance is a function of reduced cisplatin-DNA adducts, we performed DNA immuno-dot blot assays using an antibody that specifically recognizes the 1,2-d(GpG) cisplatin-DNA adduct. The 1,2(d(GpG)-cisplatin adduct represents the most prevalent adduct formed *in vitro*, in cells, and in patients treated with cisplatin (38, 39). A431 Parental (cisplatin sensitive) and A431 Pt (cisplatin resistant) cells were treated with increasing concentrations of cisplatin for 2 h, followed by PBS wash and incubation in cisplatin-free culture medium for 48 h (40, 41). Genomic DNA was then extracted and analyzed in technical duplicates to measure adduct levels (Fig. 1*A*). Using this approach, the measured levels of cisplatin–DNA adducts reflect the combined effects of cellular uptake, efflux, DNA reactivity, and repair. Both cell lines showed a dose-dependent increase in cisplatin-DNA adduct formation, while Pt cells had significantly lower levels of 1,2-d(GpG) cisplatin-DNA adducts than parental controls (Fig. 1, *A* and *B*). To confirm this finding was not cell line specific, we repeated the experiment using intrinsically cisplatin-resistant JHU006 and cisplatin-sensitive JHU029 cells treated with cisplatin. Similarly significant dose-dependent increases in 1,2-d(GpG) cisplatin-DNA adduct levels were observed with JHU006 cells consistently showing fewer adducts than JHU029 cells (Fig. 1, *C* and *D*). Uniform DNA loading was confirmed using SYBR Gold (Fig. 1, *A* and *C*). These results indicate that cisplatin resistance is associated with reduced cisplatin-DNA adduct levels in both the acquired and intrinsically resistant lines used in this study.

### TIP60 knockdown increases 1,2-d(GpG) cisplatin-DNA adduct levels

**Figure 2:**
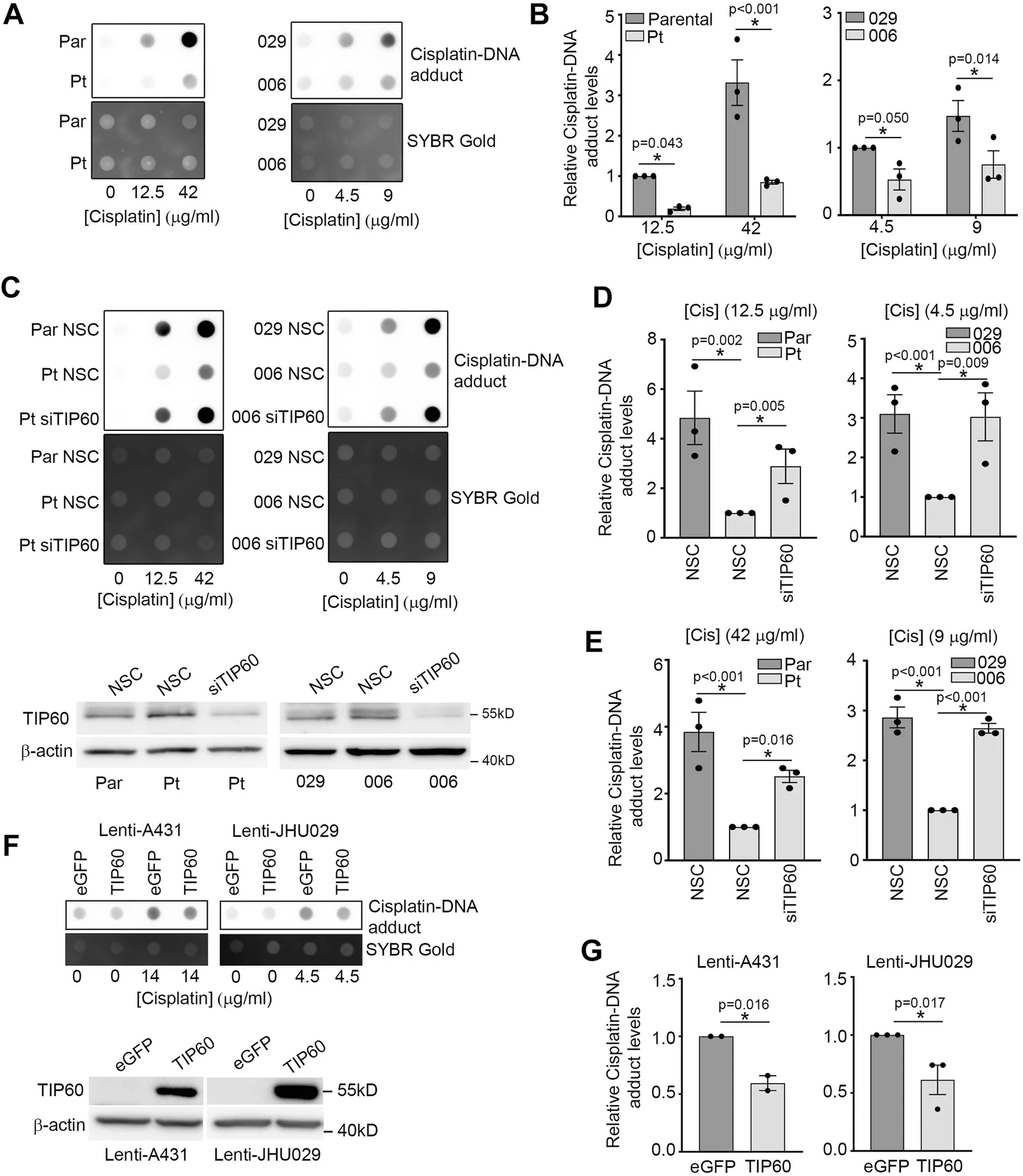
**Knockdown of TIP60 increases 1,2-d(GpG) cisplatin-DNA adduct levels.***A*, A431 Parental and Pt and JHU029 and JHU006 cells were pulsed with 12.5 μg/ml (42 μM) or 42 μg/ml (140 μM) for A431; 4.5 μg/ml (15 μM) or 9 μg/ml (30 μM) for JHU) for 2 h washed with PBS and genomic DNA was extracted immediately (time 0 h). Immuno-dot blots of genomic DNA were probed with an antibody that specifically recognizes 1,2 d(GpG) cisplatin DNA adducts and SYBR Gold to confirm equal DNA loading. *B*, densitometric quantitation with Y-axis showing cisplatin-DNA adduct levels relative to sensitive controls treated with lower doses of cisplatin (from *panel A).* Measured signal represents combined effects of uptake, efflux, and DNA reactivity during the treatment window. *C*, A431 Pt and JHU006 cells were transfected with non-silencing control (NSC) or si-RNA against TIP60, pulsed with the indicated cisplatin dose for 2 h as in *panel A*, and genomic DNA harvested immediately. Immuno-dot blots of genomic DNA were probed as in *panel A*. Non-silencing control (NSC) transfected cells served as controls. Immunoblot analysis (*bottom*) probed with TIP60 antibody and β-actin to confirm equivalent protein loading. *D, E*, densitometric quantitation of adduct levels in TIP60 knockdown cells relative to controls at (*D*) the lower doses of cisplatin (12.5 μg/ml (42 μM) and 4.5 μg/ml (15 μM) and (*E*) the higher doses of cisplatin (9 μg/ml (30 μM) and 42 μg/ml (140 μM). (*F*, *G*) Lenti-A431 cells and Lenti-JHU029 cells stably expressing eGFP (control) or TIP60 were pulsed for 2 h with vehicle or 14 μg/ml (46.5 μM) or 4.5 μg/ml (15 μM) cisplatin, respectively, and harvested 24 h later. (*F*) Immuno-dot blots of genomic DNA were probed as in *panel A*. Immunoblot analysis (*bottom*) probed with TIP60 antibody and β-actin to confirm equivalent protein loading. *G*, densitometric quantitation of adduct levels relative to lenti-eGFP controls. In *panels B*, *D*, *E*, and *G* (*right*), error bars indicate +1 S E M from three biological replicates, or from two biological replicates in *panel G* (*left*). Dose-response curves were compared using a quadratic mixed-effects model with dose-dependent variance. ∗*p* ≤ 0.05 between the best fit quadratic for each cell line.

To test whether TIP60 affects initial cisplatin-DNA adduct formation, A431 Parental (sensitive), A431 Pt (resistant), JHU029 (sensitive), and JHU006 (resistant) cells were treated with their respective IC50 doses of cisplatin (previously determined by our laboratory) for 2 h (10), followed by wash with PBS. Genomic DNA was immediately harvested to assess 1,2-d(GpG) cisplatin-DNA adducts. As expected, cisplatin-DNA adduct levels were significantly lower in Pt cells compared to parental controls at both doses (Fig. 2, *A* and *B*, *left panels*). Similarly, significantly lower levels of cisplatin-DNA adduct were observed in JHU006 compared to JHU029 at both doses (Fig. 2, *A* and *B*, *right panels*).

Next, we assessed whether TIP60 knockdown could reverse this effect. A431 Pt and JHU006 cells were transfected with TIP60 siRNA (siTIP60) or a non-silencing control (NSC), then treated with cisplatin at their respective IC50 doses for 2 h, followed by a PBS wash. Genomic DNA was immediately harvested to assess cisplatin-DNA adducts. As expected, 1,2-d(GpG) cisplatin-DNA adduct levels were lower in NSC-transfected resistant cells when compared to their sensitive counterparts (Fig. 2*C*). However, TIP60 knockdown significantly increased 1,2-d(GpG) cisplatin-DNA adduct levels in both A431 Pt and JHU006 cells at both doses, as shown in Figure 2*C* and quantitated in Figure 2, *D* and *E*. TIP60 knockdown was confirmed by immunoblotting (Fig. 2*C*, *bottom panels*). Conversely, to determine whether stable TIP60 expression reduced cisplatin-DNA adduct levels in cisplatin-sensitive cells, Lenti-A431 and Lenti-JHU029 cells stably expressing either enhanced Green Fluorescent protein (eGFP) (control) or TIP60 were treated with vehicle or their respective IC50 doses of cisplatin for 2 h (10). DNA was harvested to determine 1,2 d(GpG) adduct levels following cisplatin pulse. In both cell lines, cisplatin-DNA adducts were significantly reduced in cells stably expressing TIP60 relative to eGFP controls in Lenti-A431 and Lenti-JHU029 (Fig. 2, *F* and *G*). Stable expression of TIP60 was confirmed by immunoblotting (Fig. 2*F*, *bottom panels*). Taken together, these results indicate that TIP60 plays a role in limiting cisplatin-DNA adduct levels in cisplatin-resistant cells.

While the 1,2-d(GpG) intrastrand adducts are the most prevalent, cisplatin induces several different DNA lesions including mono adducts and interstrand crosslinks, 1,3(GpXpG) lesions (38, 39), that are not detected by the antibody-based dot-blot assays (39). To evaluate the effects of TH1834, a pharmacological inhibitor of TIP60, on total cisplatin-DNA adduct levels, we performed quantitative ICP-MS analysis in A431-Par and A431-Pt cells following the same 2-h cisplatin pulse exposure used in DNA adduct experiments, with subsequent incubation in media containing vehicle or the TIP60 inhibitor TH1834 (Fig. S1*A*). ICP-MS measurements paralleled dot-blot measurements, with total cisplatin-DNA adduct levels being lower in the resistance cells as expected with 1,2-d(GpG) driving 65% of the signal from the ICP-MS determination of total cisplatin-DNA adduct levels. This finding indicates that our dot-blot assays provide a reliable measure of the dominant form of DNA damage and repair in this model. Also, post-pulse treatment with TH1834 increased cisplatin-DNA adduct levels, suggesting that TH1834 enhances cisplatin sensitivity, potentially by impairing DNA repair and/or promoting intracellular drug retention.

### ABCC1 is overexpressed and correlates with TIP60 levels in cisplatin-resistant cells

**Figure 3:**
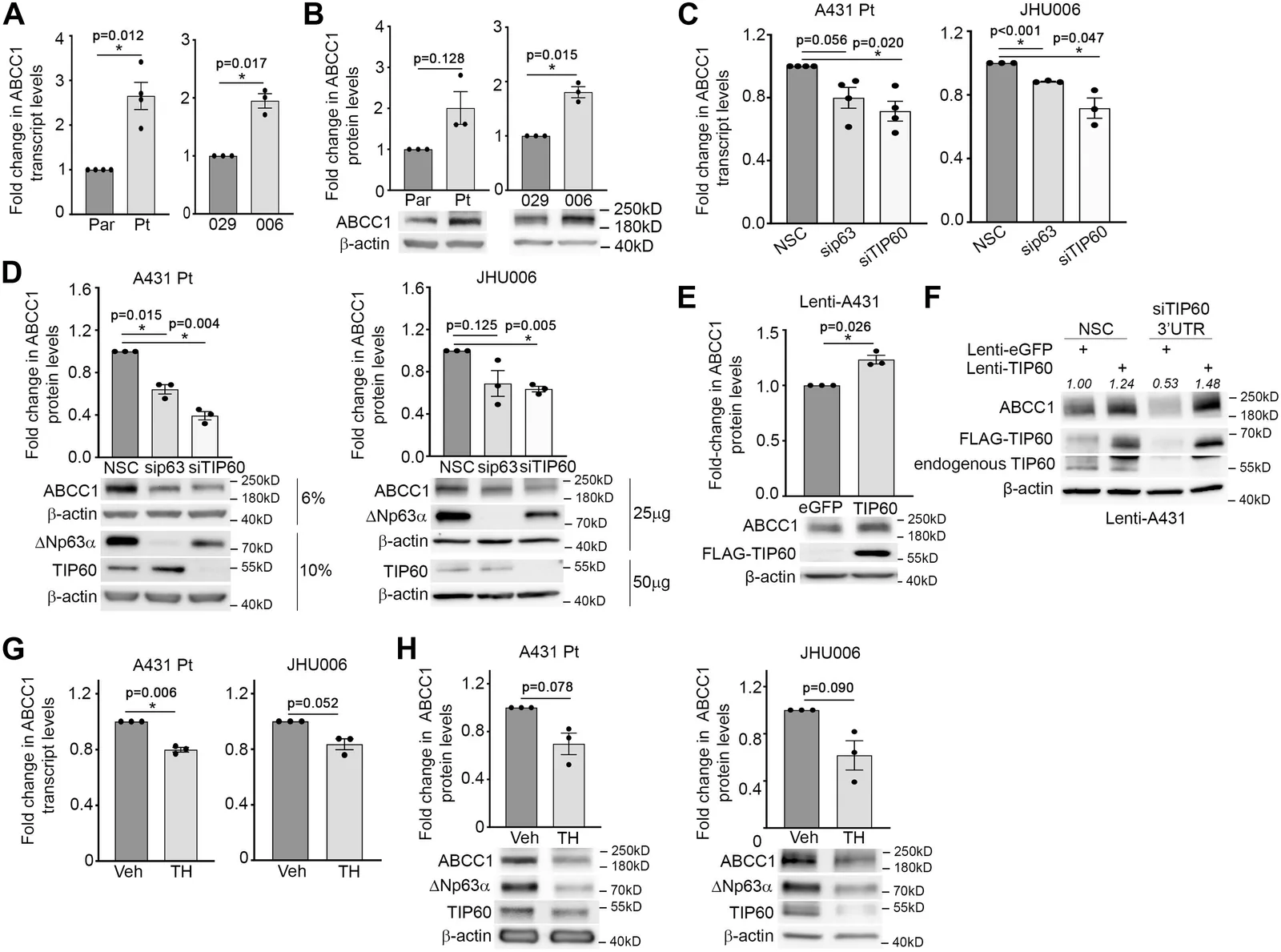
**TIP60 regulates ABCC1 levels in cisplatin resistant cells.***A*, qRT-PCR of ABCC1 transcript levels in A431 Parental and Pt cells and JHU029 and JHU006 cells. The y-axis indicates the fold change relative to Parental or JHU029 cells. *B*, immunoblots and densitometry of ABCC1 protein levels normalized to β-actin in A431 and JHU cells. *C*, qRT-PCR of ABCC1 transcript levels at 48 h post transfection from A431 Pt and JHU006 cells transfected with non-silencing control (NSC) or sip63 or siTIP60. *D*, immunoblot analysis of ABCC1, ΔNp63α and TIP60 in cells transfected with NSC, sip63 or siTIP60 at 48 h post-transfection. Densitometric analysis was performed to determine the fold change in ABCC1 levels relative to NSC after normalization to β-actin. *E*, lentiviral transduced A431 cells stably expressing eGFP (control) or TIP60 were harvested 24 h later for immunoblot analysis of TIP60 and ABCC1. Densitometric analysis was performed to determine the fold change in ABCC1 levels relative to lenti-eGFP after normalization to β-actin. *F*, Lenti-A431 cells stably expressing eGFP (control) or Flag-TIP60 were transiently transfected with NSC (control) or siRNA to the 3′UTR of TIP60 to silence endogenous TIP60. Cells were harvested 48 h later for immunoblot analysis of TIP60 and ABCC1. Densitometric analysis indicating the fold change in ABCC1 levels relative to lenti-eGFP after normalization to β-actin is shown above the ABCC1 blot. Portions of this panel (immunoblots for β-actin loading control, endogenous-Tip60 and Flag-TIP60) are shared with Figure 7*F*, as both figures derive from the same experiment. *G*, A431 Pt and JHU006 cells were treated with 50 μM of TH1834 or vehicle (DMSO) for 24 h qRT-PCR of ABCC1 transcript levels relative to vehicle treated controls. *H*, immunoblots and densitometry from three biological replicates of ABCC1 protein levels normalized to β-actin in A431 and JHU cells treated with 50 μM TH1834 *versus* vehicle controls. All RT-PCR experiments (*panels A*, *C*, *G*) were run in technical triplicate. In all panels: Error bars indicate as +1 S E M. from three biological replicates. ∗*p* ≤ 0.05 calculated using one-sample t-tests. “ns” indicated non-significance (*p* > 0.05).

ABC family exporter proteins reduce intracellular drug levels by promoting drug efflux, thereby diminishing chemotherapy efficacy. Among the ATP-binding cassette transporters, ABCC1 is highly expressed in head and neck squamous cell carcinoma (HNSCC) and is strongly associated with chemoresistance (22, 23). To evaluate the role of ABCC1 in cisplatin resistance, we measured the expression of ABCC1 and its impact on cisplatin-DNA adduct levels in cisplatin-sensitive and resistant cells. ABCC1 transcript levels were significantly upregulated in cisplatin-resistant Pt and JHU006 cells compared to their respective Parental and JHU029 sensitive controls (Fig. 3*A*). Consistent with these data, ABCC1 protein levels were also higher in resistant cells compared to sensitive cells (Pt *versus* Par and JHU006 *versus* JHU029) (Fig. 3*B*). These results suggest that TIP60 may promote cisplatin resistance by upregulating ABCC1 expression.

Given the role of ABCC1 as a drug efflux transporter, these findings predict corresponding differences in intracellular cisplatin levels. To assess platinum influx and efflux, we performed a time-course experiment using ICP-MS to assess total cellular cisplatin. During the drug incubation phase, which assessed the balance of influx and efflux, a typical drug accumulation response was observed with A431 Pt cells exhibiting consistently lower intracellular cisplatin levels compared to parental cells (Fig. S1*B*). After drug removal and replacing with media devoid of cisplatin, we assessed efflux over time. The results demonstrated that the parental cells displayed relatively minimal efflux, while cisplatin levels in Pt cells remained low, consistent with the cisplatin sensitivity profiles for these cell lines. Resulting temporal accumulation profiles suggest that drug influx is similar between the cell lines, as an alteration in influx would have resulted in the parental cell not exhibiting a plateau during the drug accumulation phase. Importantly, the data also suggest that Pt cells have increased efflux compared to the parental cell lines, consistent with the increased expression of ABCC1.

### ABCC1 expression is regulated by the ΔNp63α/TIP60 axis and inhibited by TH1834

Previous studies have shown that ΔNp63 positively regulates ABCC1 transcript levels in HNSCC (22, 24). Given that TIP60 positively regulates ΔNp63α, we investigated whether TIP60 also regulates ABCC1 transcript levels in cisplatin resistant cells. A431 Pt and JHU006 cells were transfected with siRNA targeting p63 (sip63) or TIP60 (siTIP60). Knockdown of either ΔNp63α or TIP60 reduced ABCC1 transcript levels in both cell lines (Fig. 3*C*). Consistently, ΔNp63α or TIP60 knockdown reduced ABCC1 protein levels in both A431 Pt and JHU006 cells (Fig. 3*D*). Although the difference at times did not reach statistical significance, reduced ABCC1 expression was observed across all biological replicates. TIP60 and ΔNp63α knockdown was confirmed by immunoblot (Fig. 3*D*, *bottom panels*) in both the cell lines. Consistent with this observation, TIP60 knockdown significantly decreased ABCC1 protein levels relative to NSC controls in Detroit-562, another cisplatin resistant HNSCC cell line confirming that the effects are not cell-line specific (Fig. S2, *A* and *B*). Conversely, stable expression of Flag-tagged TIP60 in lenti-A431 cells significantly increased ABCC1 protein levels relative to Lenti-eGFP controls (Fig. 3*E*).

To rule out off-target knockdown effects and directly test whether ABCC1 regulation is dependent on TIP60, we performed a rescue experiment in lenti-A431 cells stably expressing either eGFP or TIP60. In cells transfected with NSC, stable TIP60 expression increased ABCC1 levels relative to the eGFP control (Fig. 3*F*). To selectively deplete endogenous TIP60 while preserving exogenous expression, we employed an siRNA targeting the TIP60 3′UTR. Silencing endogenous TIP60 reduced ABCC1 expression in eGFP-expressing cells (Fig. 3*F*). Importantly, TIP60 overexpression rescued ABCC1 levels in cells with depleted endogenous TIP60 (Fig. 3*F*), confirming that ABCC1 regulation is dependent on TIP60.

To further assess the role of TIP60 acetyltransferase activity in the regulation of ABCC1, we employed TH1834, a pharmacological inhibitor of TIP60 acetyltransferase activity. Although TH1834 has been proposed to bind the acetyl-CoA binding domain of TIP60, direct evidence of target engagement has not yet been reported (42, 43, 44, 45, 46). To determine whether TH1834 physically interacts with TIP60 in a cellular context, we performed a cellular thermal shift assay (CETSA), a biophysical approach that uses ligand-induced thermal stabilization of proteins as evidence of drug-target binding (47, 48). CETSA was first performed using soluble lysates from lenti-A431 cells stably overexpressing TIP60. Treatment with 50 μM TH1834 produced a + 7 °C shift in the thermal denaturation curve of TIP60 relative to vehicle control (Fig. S3*A*), consistent with ligand-induced stabilization. To determine whether TH1834 also engages endogenous TIP60, CETSA was performed using lysates from JHU006 cells. Lysates incubated with 100 μM TH1834 produced a +5 °C shift in the thermal denaturation curve of endogenous TIP60 relative to vehicle control (Fig. S3*B*). To evaluate assay specificity, we examined β-actin, a housekeeping protein not expected to interact with TH1834. TH1834 treatment did not alter the levels of β-actin under either condition, confirming that the observed thermal shift was specific to TIP60 (Fig. S3, *A* and *B*). These data provide direct evidence that TH1834 engages both overexpressed and endogenous TIP60 in cellular lysates, supporting the interpretation that the phenotypes observed upon TH1834 treatment in this study are mediated through on-target inhibition of TIP60 acetyltransferase activity.

We next treated A431 Pt and JHU006 cells with 50 μM TH1834. TH1834 treatment decreased ABCC1 transcript levels in A431 Pt and JHU006 cells (Fig. 3*G*). TH1834 treatment also reduced ABCC1 transporter protein levels in both A431 Pt and JHU006) cells (Fig. 3*H*). Taken together, these results support a novel mechanism in which TIP60 either directly or through ΔNp63α enhances ABCC1 expression, thereby potentially contributing to reduced intracellular cisplatin accumulation and promoting cisplatin resistance.

### ABCC1 contributes to reduced 1,2-d(GpG) cisplatin-DNA adduct levels in resistant cells

**Figure 4:**
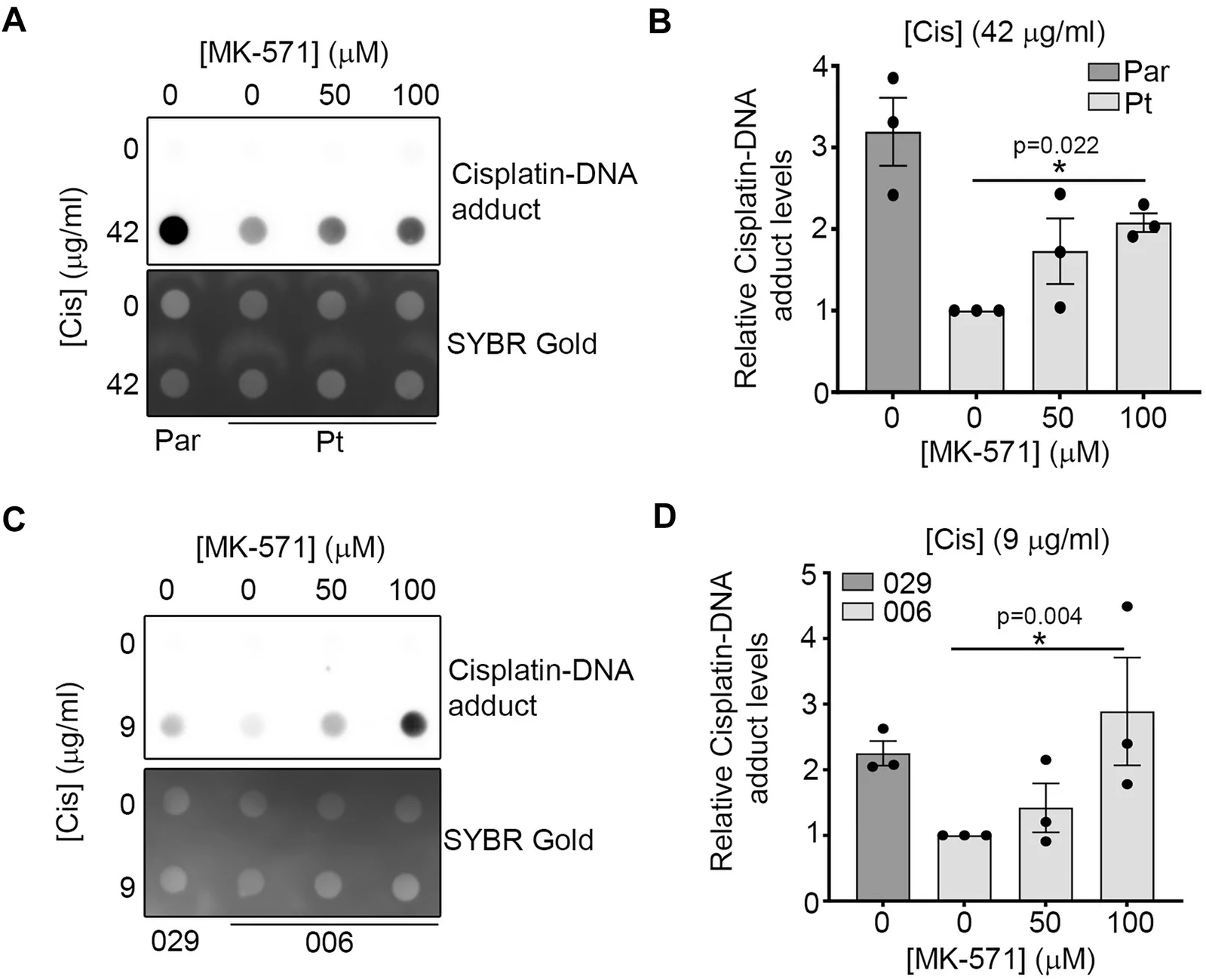
**Inhibition of ABCC1 increases 1,2-d(GpG) cisplatin-DNA adduct levels in cisplatin resistant cells.***A, C*, A431 Pt and JHU006 cells pre-treated with vehicle or MK-571 for 24 h, then pulsed with cisplatin for 2 h (42 μg/ml (140 μM) for A431; 9 μg/ml (30 μM) for JHU006), washed with PBS and harvested immediately for genomic DNA isolation. Immuno-dot blots of genomic DNA were probed with an antibody that specifically recognizes 1,2 d(GpG) cisplatin DNA adducts and SYBR Gold staining used to confirm equal DNA loading. *B, D*, densitometric quantitation with the y-axis indicating adduct levels relative to vehicle treated Parental cells (*B*) or and JHU029 (*D*) cells. The measured signal represents cisplatin-DNA adduct levels following pulse exposure, reflecting combined effects of uptake, efflux, and DNA reactivity during the treatment window. Error bars indicate +1 S E M from three biological replicates; ∗*p* ≤ 0.05 calculated using linear mixed-effects models or one- or two-sample t-tests, as appropriate.

To examine whether ABCC1 mediates cisplatin-DNA adduct suppression, we first assessed whether ABCC1 knockdown increases the 1,2-d(GpG) cisplatin-DNA adduct levels. A431 Pt-resistant cells were transfected with ABCC1 siRNA or a non-silencing control (NSC), then treated with cisplatin at 42 μg/ml for 2 h, followed by a PBS wash. Genomic DNA was immediately harvested to assess cisplatin-DNA adduct levels. As expected, NSC-transfected Pt cells exhibited fewer adducts than their sensitive Parental counterparts. Knockdown of ABCC1 resulted in a trend towards increased adduct levels in A431 Pt cells (Fig. S4, *A* and *B*). Next, A431 Pt cells and JHU006 cells were pre-treated with the selective ABCC1 inhibitor MK-571 or vehicle for 24 h prior to cisplatin treatment (42 μg/ml for A431 Pt and 9 μg/ml for JHU006) for 2 h, followed by a PBS wash. Cells were then harvested immediately following the cisplatin pulse to quantify cisplatin-DNA adduct levels. As expected, resistant cells formed fewer adducts than their sensitive controls. Interestingly, MK-571 treatment significantly increased cisplatin-DNA adduct levels in both A431 Pt (Fig. 4, *A* and *B*) and JHU006 cells (Fig. 4, *C* and *D*). These results suggest that ABCC1 contributes to limiting cisplatin-DNA adduct levels in cisplatin-resistant cells. Taken together, these results indicate that increased ABCC1 levels contribute to cisplatin DNA adduct levels and resistance, and that TIP60 may mediate this effect through ABCC1 regulation.

### Pharmacological inhibition of TIP60 increases 1,2-d(GpG) cisplatin-DNA adduct levels

**Figure 5:**
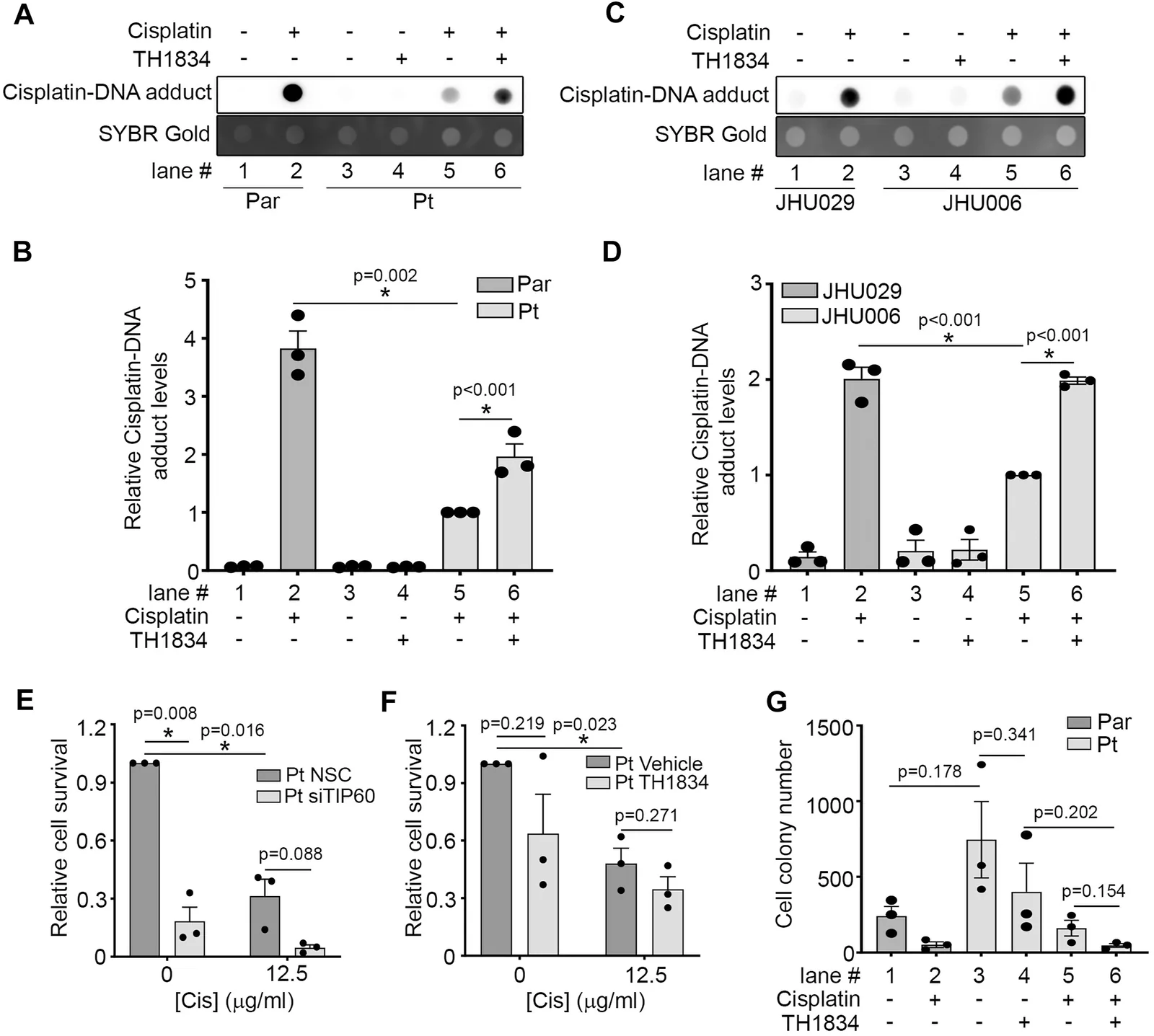
**TIP60 inhibition increases 1,2-d(GpG) cisplatin-DNA adduct persistence and reduces survival of resistant cells.***A, C*, A431 Parental and Pt and JHU029 and JHU006 cells pulsed with cisplatin for 2 h (42 μg/ml for A431; 9 μg/ml for JHU), washed with PBS and subsequently maintained in cisplatin-free medium containing vehicle (DMSO) or 50 μM TH1834 for an additional 24 h. Immuno-dot blots of genomic DNA were probed with an antibody that specifically recognizes 1,2-d(GpG) cisplatin DNA adducts and SYBR Gold staining used as DNA loading control. *B, D*, densitometric quantitation from Panels *A* and *C* with the y-axis indicating cisplatin-DNA adduct levels relative to cisplatin-treated resistant cells. The measured signal represents cisplatin-DNA adduct levels following pulse exposure, reflecting combined effects of uptake, efflux, and DNA reactivity during the treatment window. *E*, cell survival assay of A431 Pt cells transiently transfected with siTIP60 or non-silencing control (NSC) and pulsed with vehicle or 12.5 μg/ml cisplatin for 2 h, washed in PBS and returned to complete medium. The y-axis shows cell survival relative to Pt NSC vehicle. (*F*) Cell survival assay of A431 Pt cells pulsed with vehicle or 12.5 μg/ml cisplatin for 2 h and treated with vehicle (DMSO) or 50 μM TH1834. The y-axis shows cell survival relative to vehicle. *G*, A431 Pt were grown in soft agar, pulsed for 2 h with 6.25 μg/ml cisplatin (half the IC50 dose) followed by continuous treatment with vehicle (DMSO) or 50 μM TH1834. Colonies were stained after 21 days and quantified by ImageJ. The y-axis indicates colony number. In *panels B*-*G*: Error bars indicate +1 S E M from three biological replicates; ∗*p* ≤ 0.05 calculated using linear mixed-effects models or one- or two-sample t-tests, as appropriate. “ns” indicated non-significance (*p* > 0.05).

While TIP60 is known to play a role in DNA damage response and repair (49), its impact on 1,2-d(GpG) cisplatin-DNA adduct levels has not been previously reported. We therefore investigated whether the catalytic activity of TIP60 promotes the repair of cisplatin-induced DNA adducts. A431 Pt cells were treated with 42 μg/ml cisplatin (IC50) for 2 h, followed by PBS wash and incubation in cisplatin-free medium with either vehicle or 50 μM TH1834. A431 Parental cells treated with cisplatin served as a control. Cells were harvested 24 h later to assess remaining 1,2-d(GpG) cisplatin-DNA adduct levels. As expected, cisplatin-resistant A431 Pt cells showed significantly fewer adducts than Parental controls (*lane 5 versus 2*) and no adducts were observed in vehicle-treated controls for either cell line *(lanes one and 3)* (Fig. 5, *A* and *B*), or upon treatment with TH1834 alone (no cisplatin) (lane 4). However, TH1834 treatment following cisplatin exposure significantly increased adduct levels in A431 Pt cells compared to cisplatin alone (*lane 6 versus 5*) (Fig. 5, *A* and *B*). Similar results were obtained in naturally cisplatin-resistant JHU006 cells treated with 9 μg/ml cisplatin and TH1834 (*lane 6 versus 5*) (Fig. 5, *C* and *D*).

### TIP60 inhibition reduces survival of cisplatin-resistant cells

To evaluate the role of TIP60 in the survival of cisplatin resistant cells, cell survival assays were performed in A431 Pt cells transiently transfected with siTIP60 or NSC control and pulsed with vehicle or 12.5 μg/ml cisplatin. TIP60 knockdown reduced cell survival in vehicle treated cells and showed a similar trend in cisplatin treated cells *(lane 3 versus 4)* (Fig. 5*E*). To determine if cell survival was affected by inhibition of TIP60 activity, parallel experiments were performed in which TIP60 was inhibited pharmacologically with 50 μM TH1834 followed by a pulse with vehicle or 12.5 μg/ml cisplatin. Cell survival decreased in response to cisplatin treatment alone and showed a similar trend following TH1834 treatment. Combined treatment with cisplatin and TH1834 further reduced survival relative to cisplatin alone (*lane 4 versus 3)* (Fig. 5*F*). In 3D anchorage-independent colony formation assays, cisplatin-resistant A431 Pt cells formed more colonies than A431 Parental cells *(lane 1 versus 3)* (Fig. 5*G*). Combination treatment with TH1834 and cisplatin further reduced colony formation in A31 Pt resistant cells compared to TH1834 alone (*lane 4 versus 6*) or cisplatin alone (*lane 5 versus 6*) (Fig. 5*G*). Together, these data indicate that inhibition of TIP60 increases cisplatin-DNA adduct levels and is associated with reduced survival of cisplatin-resistant cells.

### Cisplatin-resistant cells exhibit enhanced removal of 1,2-d(GpG) cisplatin-DNA adducts

Given the known roles of ΔNp63α and TIP60 in regulating DDR genes, we next examined whether cisplatin-resistant cells differed in their ability to remove cisplatin-DNA adducts following cisplatin pulse treatment. Cisplatin induces DNA adducts rapidly, typically within 1 to 2 h of exposure, and the persistence of these adducts reflects the efficiency of cellular repair processes (50). To evaluate the persistence of cisplatin-DNA adducts after a cisplatin pulse, A431 Parental and Pt cells were treated with either 12.5 μg/ml or 42 μg/ml cisplatin for 2 h, followed by PBS wash and incubation in cisplatin-free medium. The 2-h post-treatment time point was defined as “time 0” and genomic DNA was isolated at the indicated time points to measure remaining cisplatin-DNA adducts levels relative to this baseline. At the lower dose, Pt cells showed a significantly greater reduction in cisplatin-adduct levels over time when compared to Parental cells (Fig. S5*A*, *left panel* and Fig. S5*B*). At the higher dose, Parental cells retained relatively high levels of cisplatin-DNA adducts, whereas the Pt cells efficiently repaired adducts over time (Fig. S5*A, right panel* and Fig. S5*C*, compared to A431 Par). These results indicate that cisplatin-resistant A431 Pt cells have an enhanced ability to remove cisplatin-DNA adducts and thereby DNA repair capacity compared to their cisplatin-sensitive counterparts.

### Pharmacological inhibition of TIP60 impairs removal of 1,2-d(GpG) cisplatin-DNA adducts

To determine whether TIP60 contributes to removal of cisplatin-DNA adducts, we examined the effect of pharmacological inhibition of TIP60 acetyltransferase activity on cisplatin DNA adduct levels in A431 Pt cells. Cells were pulsed with 42 μg/ml cisplatin (IC50) for 2 h, followed by a PBS wash and incubation in medium with either vehicle or 50 μM TH1834. Genomic DNA was harvested at the indicated time points to measure DNA adduct levels relative to baseline (0 h after pulse). A431 Pt cells treated with TH1834 retained significantly higher levels of cisplatin-DNA adducts over time compared with vehicle-treated controls (*i.e.*, cisplatin alone, no TH1834 (Fig. S6, *A* and *B*). These results suggest that TIP60 activity contributes to the removal of cisplatin-induced DNA adducts following cisplatin exposure.

### TIP60 regulates DNA damage response gene expression in cisplatin-resistant cells

**Figure 6:**
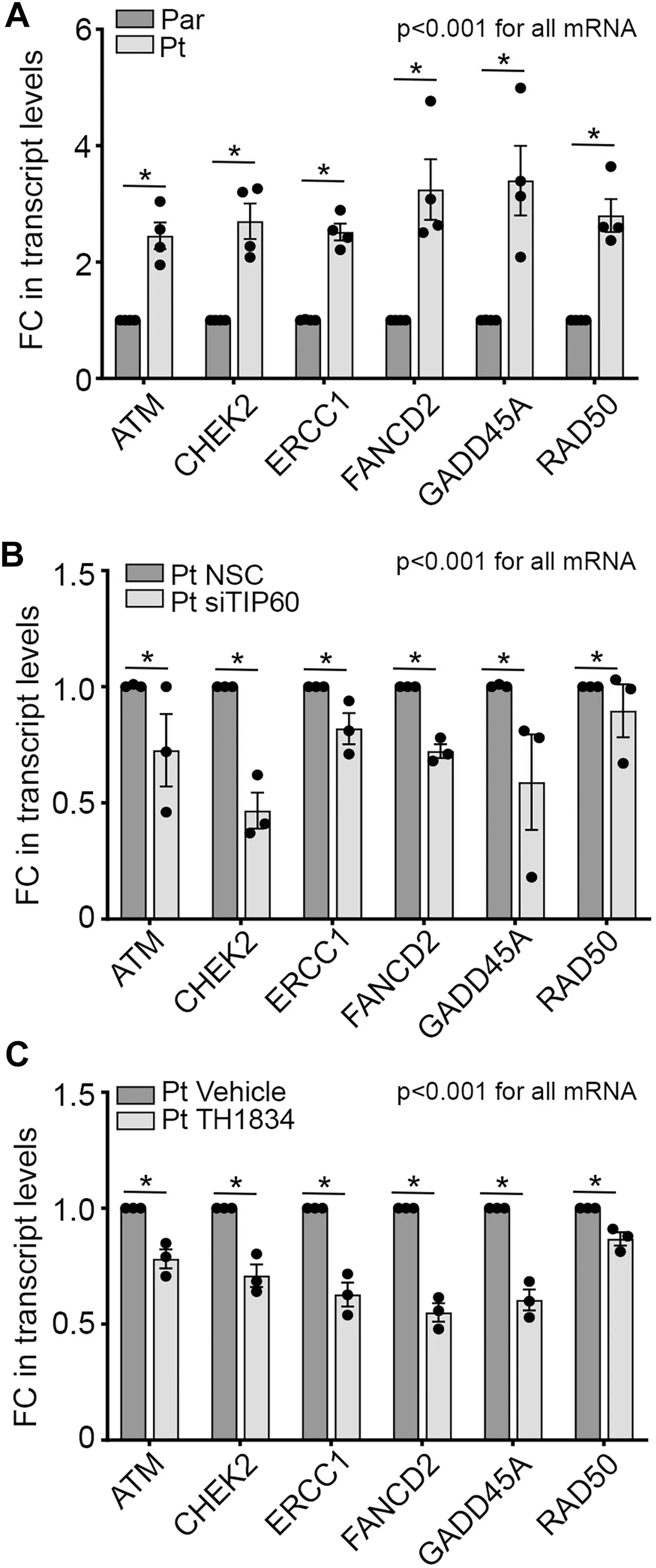
**TIP60 regulates the expression of DNA damage response genes in cisplatin resistant cells.** Transcript levels of the indicated DNA Damage response (DDR) genes transcript levels were measured in (*A*) A431 Parental and Pt, (*B*) A431 Pt cells transfected with NSC or siTIP60 and (*C*) A431 Pt cells treated with either vehicle (DMSO) or 50 μM TH1834. Cells were harvested at 48 h post transfection or 24 h after TH1834 treatment. The y-axis indicates fold change in transcript levels relative to (*A*) Parental, (*B*) Pt NSC, or (*C*) Pt Vehicle. In all panels: Error bars indicate +1 S E M from three biological replicates; ∗*p* ≤ 0.05 calculated by one-sample *t* test.

Cisplatin resistance has been linked to altered expression of genes involved in the DDR (26). Both ΔNp63α and TIP60 are known to regulate DDR genes (8, 13, 28, 29) and our prior work demonstrated that TIP60 stabilizes and acetylates ΔNp63α, with both proteins correlating with cisplatin resistance. To investigate the role of the ΔNp63α/TIP60 axis in DDR gene regulation, we screened 60 DDR-related genes (Table S1) and compared transcript levels between A431 Parental and Pt cells. Transcript levels of several genes, including CHEK2, CDKN1A, RAD51B, RAD18, and RAD21 were upregulated in A431 Pt cells relative to Parental controls. mRNAs functionally linked to ATM/ATR signaling were prioritized for validation, as this pathway constitutes a primary regulatory axis of the DDR activated by cisplatin-induced DNA lesions. Increases in ATM, CHEK2, ERCC1, FANCD2, GADD45A, and RAD50 in Pt cells relative to parental controls were validated by Taq-Man-based qRT-PCR (Fig. 6*A* for all transcripts), confirming their association with acquired resistance. To assess whether TIP60 regulates these genes, we performed TIP60 knockdown in A431 Pt cells. Transcript levels of the DDR genes decreased significantly relative to the non-silencing control (NSC) (Fig. 6*B* for all mRNA). Pharmacological inhibition of TIP60 with TH1834 similarly decreased expression of these genes (Fig. 6*C* for all mRNA), suggesting these genes function downstream of TIP60 signaling in cisplatin-resistant cells. Together, these results indicate that TIP60 promotes cisplatin resistance by enhancing expression of DDR genes, thereby supporting cellular survival following DNA damage.

### TIP60 and ΔNp63α regulate XPC expression and NER-associated removal of 1,2-d(GpG) cisplatin-DNA adducts in cisplatin-resistant cells

**Figure 7:**
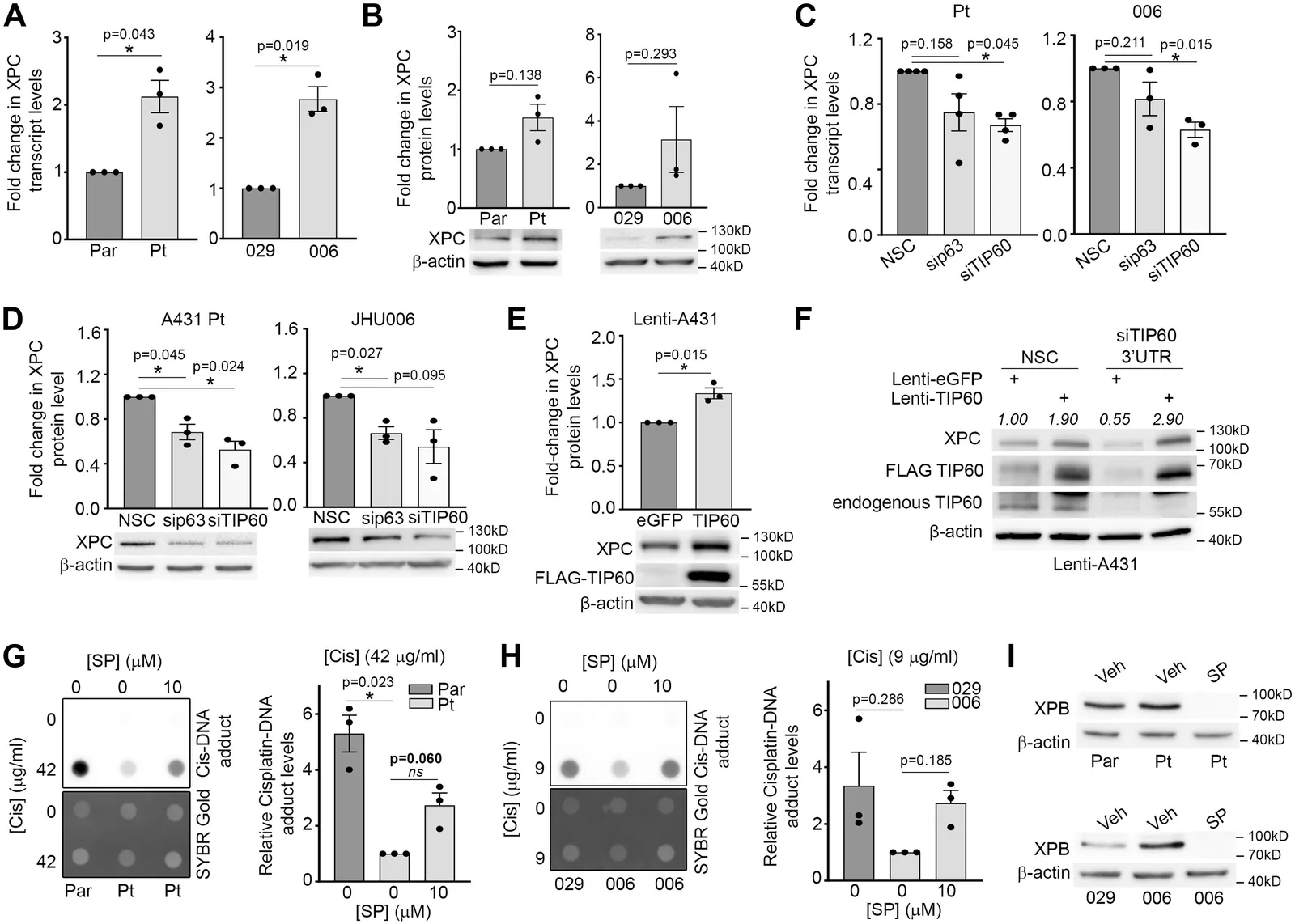
**TIP60 regulates XPC levels and modulates NER in cisplatin resistant cells.***A*, qRT-PCR of XPC transcript levels in A431 Parental *versus* Pt cells and JHU029 *versus* JHU006 cells. The y-axis indicates fold change in XPC transcript levels relative to Parental or JHU029 cells. *B*, immunoblot analysis of XPC protein levels A431 Parental *versus* Pt or JHU029 *versus* JHU006 cells and densitometric analysis normalized to β-actin. The y-axis indicates the fold change in XPC protein levels relative to Parental or JHU029 cells. (*C*, *D*) A431 Pt and JHU006 cells transfected with NSC, sip63 or siTIP60 were harvested at 48 h. *C*, XPC transcript levels were measured by qRT-PCR. The y-axis indicates the fold change in the transcript levels relative to Pt NSC or JHU006 NSC. *D*, immunoblot and corresponding densitometric analysis with y-axis showing the fold change in XPC protein relative to NSC. *E*, lentiviral transduced A431 cells stably expressing eGFP (control) or TIP60 were immunoblotted for TIP60, XPC and β-actin. Densitometric analysis was performed to determine the fold change in XPC levels relative to lenti-eGFP after normalization to β-actin. *F*, Lenti-431 cells stably expressing eGFP (control) or Flag-TIP60 were transiently transfected with NSC or siRNA targeting to the 3′UTR of TIP60 to silence endogenous TIP60. Cells were harvested 48 h later for immunoblot analysis of TIP60 and XPC. Densitometric analysis indicating the fold change in XPC levels relative to lenti-eGFP after normalization to β-actin is shown above the XPC blot. Portions of this panel (immunoblots for β-actin loading control, endogenous-Tip60 and Flag-TIP60) are shared with Figure 3*F*, as both figures derived from the same experiment. *G*, *H*, (*G*) A431 Pt and (*H*) JHU006 cells were pretreated with vehicle or Spironolactone (SP) for 2 h, pulsed with cisplatin (42 μg/ml for A431 and 9 μg/ml for JHU), washed in PBS, and subsequently maintained in cisplatin-free medium until harvest 24 h later. Immuno-dot blot of genomic DNA probed with an antibody that specifically recognizes 1,2 d(GpG) cisplatin DNA adducts and SYBR Gold staining was used to confirm equal DNA loading. The y-axis indicates cisplatin-DNA adduct levels relative to resistant control cells. The measured signal represents cisplatin-DNA adduct levels following pulse exposure, reflecting combined effects of uptake, efflux, and DNA reactivity during the treatment window. *I*, immunoblot analysis of XPB levels in A431 Pt and JHU006 cells treated with vehicle or 10 μM Spironolactone (SP) for 24 h. A431 Parental and JHU029 cells treated with vehicle (DMSO) were used as control. β-actin was included as a loading control. Error bars indicate +1 S E M from n = 4 biological replicates in panel 7C (Pt) and n = 3 in all others; ∗*p* ≤ 0.05 calculated using one- or two-sample t-tests, as appropriate. “ns” indicated non-significance (*p* > 0.05).

To investigate whether the TIP60/ΔNp63α axis influences nucleotide excision repair (NER) in cisplatin resistant cells, we examined the expression of XPC, a key component of the NER damage recognition complex and repair initiation pathway (14, 33, 34). Cisplatin-resistant Pt cells expressed higher XPC transcript and protein levels than cisplatin-sensitive parental cells (Fig. 7, *A* and *B*, *left panels*). A similar trend was observed in resistant JHU006 relative to cisplatin-sensitive JHU029 cells (Fig. 7, *A* and *B*, *right panels*). To determine whether ΔNp63α and TIP60 regulate XPC, we silenced each gene in A431 Pt and JHU006 cells. Knockdown of TIP60 significantly decreased XPC transcript in both cell lines (Fig. 7*C*), whereas p63 knockdown also showed a trend towards decreased XPC transcript levels in both cell lines. Knockdown of either ΔNp63α or TIP60 significantly decreased XPC protein levels in Pt cells (Fig. 7*D*) while sip63 significantly reduced XPC protein in JHU006 and siTIP60 showed a similar trend. Consistent with this, TIP60 knockdown decreased XPC protein levels in Detroit-562 cells, another cisplatin-resistant HNSCC cell line, relative to NSC control, confirming that the effects are not cell-line specific (Fig. S2, *A* and *C*). Conversely, stable expression of TIP60 in lenti-A431 cells increased XPC protein levels relative to lenti-eGFP controls (Fig. 7*E*). Together, these results support a model in which both TIP60 and ΔNp63α positively regulate XPC expression in cisplatin-resistant cells.

To rule out off-target knockdown effects and directly test whether XPC regulation is dependent on TIP60, we performed a rescue experiment in lenti-A431 cells stably expressing either eGFP or TIP60. In cells transfected with NSC, stable TIP60 expression increased XPC levels relative to eGFP control, while silencing endogenous TIP60 reduced XPC expression in eGFP-expressing cells (Fig. 7*F*). Importantly, TIP60 overexpression rescued XPC levels in cells with depleted endogenous TIP60, confirming that XPC regulation is dependent on TIP60.

To determine whether XPC contributes to the removal of 1,2-d(GpG) cisplatin-DNA adducts, A431 Pt cells were transfected with siRNA targeting XPC or a non-silencing control (NSC) and treated with 42 μg/ml cisplatin for 2 h, followed by a PBS wash and genomic DNA was extracted immediately. As expected, cisplatin-DNA adduct levels were significantly lower in NSC-transfected Pt cells than in sensitive Parental counterparts. XPC knockdown trended towards an increase in cisplatin-DNA adduct levels in A431 Pt cells (Fig. S7, *A* and *B)*. These results are consistent with a role of XPC in the removal of cisplatin-DNA adducts.

To further examine the functional role of NER, we used Spironolactone (SP) to induce the degradation of XPB, an essential helicase required for NER. Since there is no direct XPC inhibitor available, SP was used to block TFIIH-dependent DNA unwinding and thereby impair NER progression without directly targeting XPC (51). A431 Pt cells and JHU006 cells were pretreated with SP for 2 h, pulsed with cisplatin (42 μg/ml or 9 μg/ml, respectively) for 2 h in the presence or absence of 10 μM spironolactone, and returned to cisplatin-free medium containing vehicle or spironolactone, after which 1,2-d(GpG) cisplatin-DNA adduct levels were measured 24 h later. As expected, resistant cells had lower adducts than their sensitive counterparts. SP-mediated depletion of XPB increased cisplatin-DNA adduct levels in both A431 Pt (Fig. 7*G* and JHU006 cells (Fig. 7*H*). Depletion of XPB following SP treatment was confirmed by immunoblot analysis (Fig. 7*I*). Collectively, these findings indicate that ΔNp63α and TIP60 contribute to cisplatin resistance, at least in part, by promoting NER-associated removal of cisplatin-DNA adducts through regulation of XPC and related repair functions.

### TH1834 treatment reduces survival and enhances cell death in cisplatin-resistant cells lacking ABCC1

**Figure 8:**
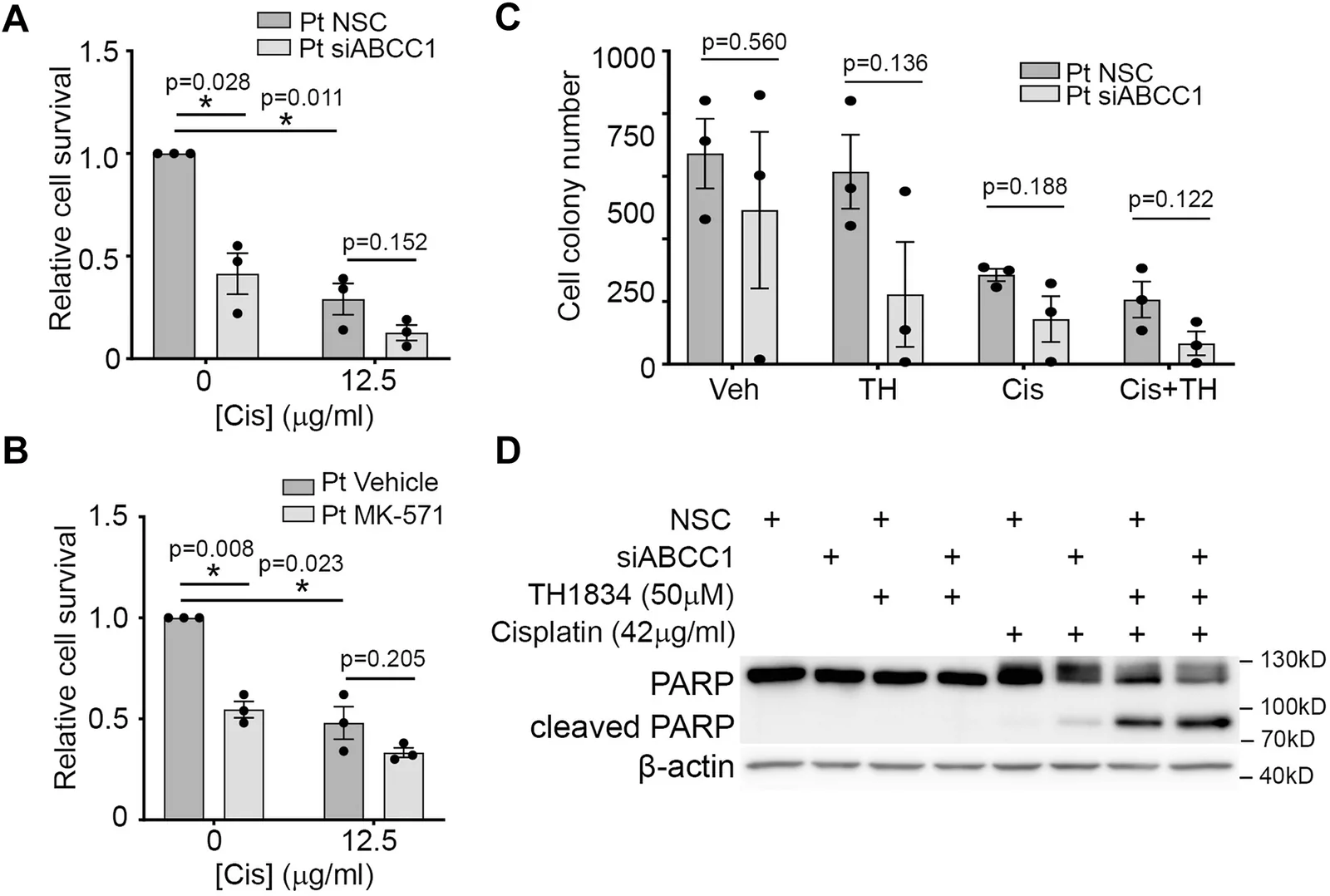
**TH1834 treatment increases cell death in ABCC1-depleted cisplatin resistant cells.***A*, survival assay of A431 Pt cells transfected with non-silencing control (NSC) and siABCC1 and plated at low density, then pulsed with vehicle or 12.5 μg/ml cisplatin for 2 h, washed with PBS, and subsequently maintained in cisplatin-free medium. Survival shown relative to Pt NSC. *B*, survival assay of A431 Pt cells pre-treated with 50 μM MK-571 for 24 h, pulsed with vehicle or 12.5 μg/ml cisplatin for 2 h, washed with PBS, and subsequently maintained in cisplatin-free medium containing either vehicle or MK-571. Survival shown relative to A431 Pt vehicle. *C*, colony formation assay of A431 Pt cells transfected with non-silencing control (NSC) and siABCC1 and grown in soft agar, pulsed for 2 h with 6.25 μg/ml (half of the IC50) cisplatin, washed with PBS, and subsequently overlayed with cisplatin-free medium containing vehicle (DMSO) or 50 μM TH1834 continuously for 21 days. Colonies were then stained and quantified by ImageJ. Y-axis indicates colony number. In *panels A*-*C*: Error bars indicate +1 S E M from three biological replicates; ∗*p* ≤ 0.05 calculated using one- or two sample t-tests, as appropriate. *D*, immunoblot of PARP and cleaved PARP levels in A431 Pt cells transfected with non-silencing control (NSC) and siABCC1 treated with either vehicle or 50 μΜ TH1834 alone and/or cisplatin (2 h, 42 μg/ml (140 μM)), washed in PBS and subsequently maintained in cisplatin-free medium for 24 h β-actin was included as a loading control for equivalent protein. “ns” indicated non-significance (*p* > 0.05).

Based on our finding that increased ABCC1 levels contribute to reduced cisplatin DNA adduct levels and resistance (Fig. 4), we sought to determine if TIP60 knockdown or inhibition could further sensitize ABCC1-depleted cells to cisplatin-mediated cell death. ABCC1 knockdown decreased cell survival in vehicle-treated A431 Pt cells, with a further reduction observed in combination with cisplatin (Fig. 8*A*, *lane 4 versus 3*). Similarly, A431 Pt cells were pre-treated with 50 μM MK-571 for 24 h, followed by treatment with either vehicle or 12.5 μg/ml cisplatin for 2 h and then treated again with MK-571 to inhibit ABCC1. Cell survival was reduced by MK-571or cisplatin treatment alone, and further reduced by combined cisplatin and MK-571 treatment (Fig. 8*B*, *lane 4 versus 3*).

We next tested whether ABCC1 knockdown enhanced the effect of cisplatin and TH1834 on anchorage independent colony formation. Although ABCC1 knockdown alone did not significantly reduce colony number (*lane 2 versus 1*), it showed a similar trend toward reduced colony number in cells treated with TH1834 alone, cisplatin alone or both cisplatin and TH1834 (Fig. 8*C*). To assess whether reduced survival was associated with increased cell death, we analyzed PARP cleavage by immunoblotting. ABCC1 knockdown alone did not induce PARP cleavage (*lane 2 and 4*) (Fig. 8*D*). However, in ABCC1 depleted cells, cisplatin alone (*lane 6*) or cisplatin and TH1834 (*lane* 8) increased cleaved PARP levels relative to Pt NSC control cells (*lane 5 and 7*) (Fig. 8*D*). These results demonstrate that blocking ABCC1 in combination with TIP60 inhibition augments cisplatin-induced toxicity in cisplatin resistant cells.

### Inhibition of both TIP60 and NER-associated repair functions increases cell death in cisplatin-resistant cells

**Figure 9:**
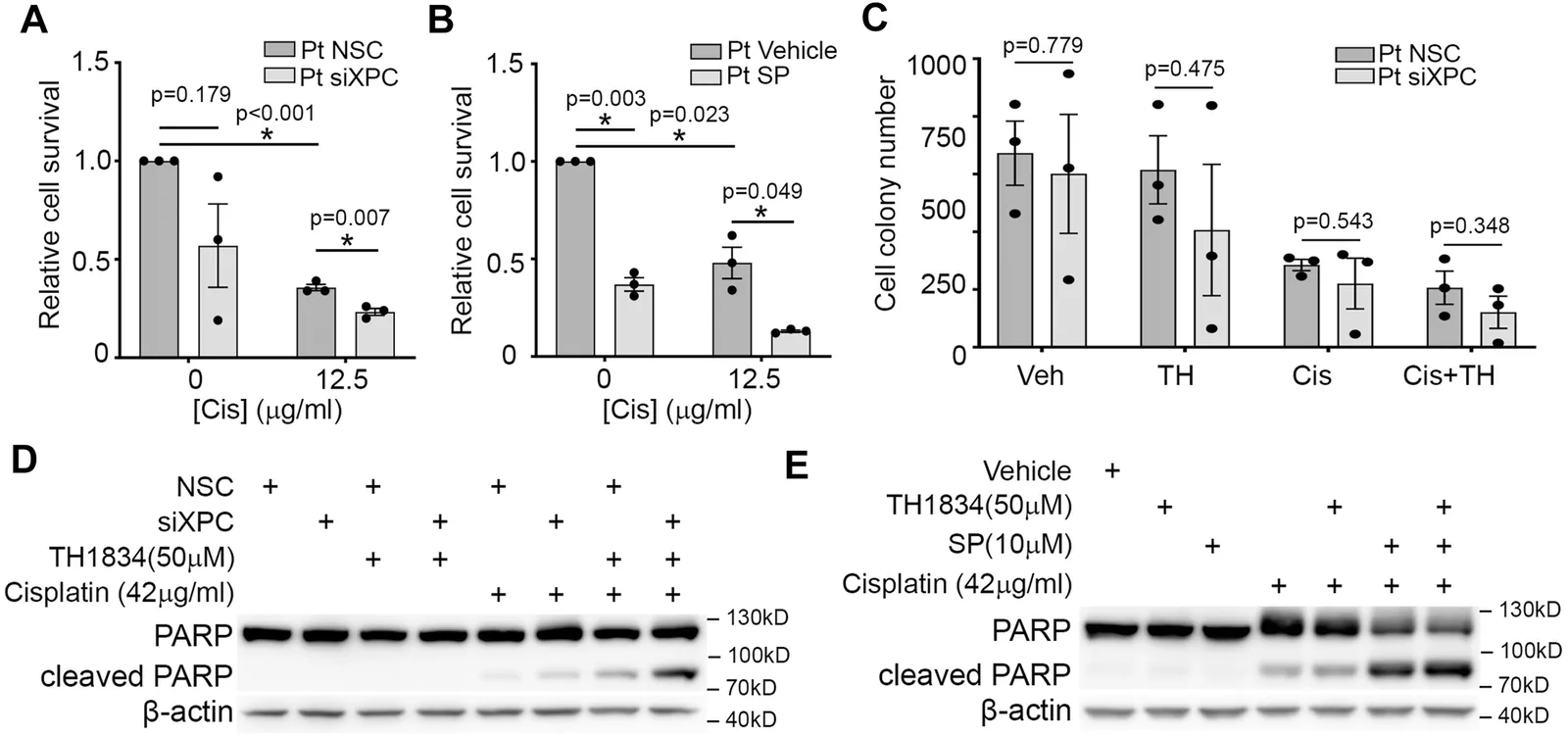
**Co-inhibition of TIP60 and NER associated repair functions increases cell death in cisplatin resistant cells.***A*, survival assay of A431 Pt cells transfected with non-silencing control (NSC) and siXPC plated at low density, pulsed with vehicle or 12.5 μg/ml cisplatin for 2 h, washed with PBS, and subsequently maintained in cisplatin-free medium. Survival shown relative to Pt NSC. *B*, survival assay of A431 Pt cells pre-treated with 10 μM SP for 24 h followed by 2 h pulse with vehicle or 12.5 μg/ml cisplatin, washed in PBS, and subsequently maintained in cisplatin-free medium with either vehicle or SP. Survival shown relative to Pt vehicle. *C*, colony formation assay of A431 Pt cells transfected with non-silencing control (NSC) and siXPC and grown in soft agar, pulsed for 2 h with 6.25 μg/ml cisplatin (half of the IC50), washed in PBS, and subsequently overlayed with cisplatin-free medium containing vehicle (DMSO) or 50 μM TH1834. Colonies were stained after 21 days and quantified by ImageJ. Y-axis indicates colony number. In panels A-C: Error bars indicate +1 S E M from three biological replicates; ∗*p* ≤ 0.05 calculated using one- or two-sample t-tests, as appropriate. *D, E*, Immunoblot of PARP and cleaved PARP levels in: (*D*) A431 Pt cells transfected with non-silencing control (NSC) and siXPC, and treated with either vehicle or 50 μΜ TH1834 alone and/or cisplatin (2 h, 42 μg/ml (140 μM), washed in PBS, and subsequently maintained in cisplatin-free medium for 24 h. *E*, A431 Pt cells pre-treated with 10 μM TH1834 alone and/or 10 μM SP for 24 h and pulsed with vehicle or 12.5 μg/ml cisplatin for 2 h, followed by washed in PBS, and subsequent maintenance in cisplatin-free medium with either vehicle, SP or TH1834. β-actin was included as a loading control. “ns” indicated non-significance (*p* > 0.05).

Building on our finding that TIP60 and ΔNp63α positively regulate the NER pathway (Fig. 7), we next examined whether perturbation of XPC, which functions in DNA damage recognition, and XPB, a helicase required downstream for NER progression, could further sensitize cells to cisplatin-mediated cell death. XPC knockdown decreased survival in vehicle-treated A431 Pt cells (Fig. 9*A*), and further reduced survival when combined with cisplatin (*lane 4 versus 3*). Next, we performed parallel experiments using SP, which causes the proteasomal-mediated degradation of XPB (51). A431 Pt cells were pre-treated with 10 μM SP for 2 h to deplete endogenous XPB, followed by pulsing with vehicle or 12.5 μg/ml cisplatin for 2 h, followed by PBS wash and subsequent SP treatment. Cell survival was significantly reduced by SP or cisplatin alone*,* with a further reduction observed upon combined cisplatin and SP treatment (Fig. 9*B, lane 4 versus 3*). To test whether XPC knockdown enhanced the effects of TIP60 inhibition, we assessed 3D colony formation in the presence of cisplatin and/or TH1834. XPC knockdown showed a similar trend toward reduced colony number across all treatments, including vehicle, TH1834 alone, cisplatin alone or both cisplatin and TH1834 (Fig. 9*C*).

To determine whether decreased cell survival reflects increased cell death, we examined PARP cleavage by immunoblotting in cells transiently transfected with siXPC or NSC control and TH1834 and/or cisplatin. XPC knockdown alone (*lane* 6) or combined with TH1834 (*lane* 8) increased cleaved PARP levels in cisplatin-treated cells relative to NSC controls (Fig. 9*D*, *lanes five and 7, respectively*). Parallel experiments were performed in cells treated with spironolactone and TH1834 and/or cisplatin. Cisplatin alone (*lane 4*) or cisplatin and TH1834 (*lane* 5) increased cleaved PARP levels (Fig. 9*E*). Importantly, cisplatin and spironolactone treatment further enhanced PARP cleavage both alone (*lane 6*) and in combination with TH1834 (Fig. 9*E*, *lane 7*). Overall, these results indicate that combined inhibition of TIP60 and the NER-associated repair functions enhances cisplatin sensitivity, leading to reduced survival and increased cell death in cisplatin-resistant cells.

Together, these results indicate that TIP60 promotes cisplatin resistance through complementary mechanisms involving reduced intracellular drug accumulation and enhanced removal of cisplatin-DNA adducts.

## Discussion

TIP60 has been increasingly recognized as a critical mediator of the DDR across multiple cancer types. Our study identifies TIP60 as a central regulator of cisplatin resistance in squamous cell carcinoma (SCC) cells, functioning through dual mechanisms (1): upregulation of the cisplatin exporter ABCC1 and thereby reducing intracellular drug levels, and (2) enhancement of nucleotide excision repair (NER) *via* transcriptional upregulation of XPC, a key DNA damage recognition protein. These complementary pathways position TIP60 as a key, though not exclusive, driver of resistance in SCC.

Cisplatin induces several types of DNA lesions, including both intrastrand and interstrand crosslinks, which differ in abundance and cytotoxicity (38, 39). Our ICP-MS studies showed that 1,2-d(Gp) intrastrand adducts were the dominant contributor to total adduct levels in our cell models, consistent with prior findings indicating intrastrand crosslinks comprise the majority of cisplatin-DNA adducts (39). Consistent with the known features of resistance, cisplatin-resistant cells exhibited reduced 1,2-d(GpG) intrastrand cisplatin-DNA adduct levels following cisplatin pulse exposure and enhanced removal of cisplatin-DNA adducts over time (52). TIP60 knockdown or inhibition increased adduct levels and impaired removal of cisplatin-DNA adducts following cisplatin pulse, demonstrating an essential role for TIP60 in protecting cells from cisplatin-induced DNA damage.

Dysregulation of drug transporters, particularly within the ABC family, is a well-established mechanism of cisplatin resistance (53, 54). ABCC1 and MDR1 have been directly linked to multidrug resistance and therapy failure (21, 55, 56). ΔNp63α has been shown to transcriptionally upregulate ABCC1, correlating with resistance in HNSCC (22, 24). TIP60 also upregulates MDR-1 expression, with clinical evidence linking MDR1 upregulation to chemoresistance (25, 57, 58). Consistent with these findings, we found elevated expressions of ABCC1 and MDR1 *(data not shown)* in resistant cells. Our data clearly showed that TIP60 inhibition or ΔNp63α/TIP60 knockdown reduced ABCC1 expression, while pharmacological inhibition of ABCC1 increased cisplatin adduct levels and reduced survival, reinforcing the contribution of TIP60/ΔNp63α-mediated drug efflux to resistance. Backed by our ICP-MS data showing that efflux profiles differ between A431 Parental and Pt cells, our findings suggest that elevated TIP60 contributes to drug resistance both by enhancing drug efflux and reducing intracellular cisplatin accumulation *via* modulation of ABCC1 expression. Taken together, our data support a model in which TH1834 potentiates cisplatin efficacy by increasing the persistence of DNA damage in resistant cells.

Although our data indicate that TIP60 and ΔNp63α are both transcriptional regulators of ABCC1, the specific mechanistic link between TIP60, ΔNp63α, and regulation of ABCC1 remains an important question. Our prior work demonstrated that TIP60 acetylates and stabilizes ΔNp63α (11). ΔNp63α has been shown to directly bind the ABCC1 locus and drive its expression (24). Together, these findings suggest a linear pathway in which TIP60 acts upstream of ΔNp63α, thereby indirectly promoting ABCC1 transcription. ΔNp63α binds within the first intron of ABCC1, serving as a direct transcriptional regulator (24). TIP60, as a chromatin acetyltransferase, is frequently recruited to active enhancers and promoters to acetylate histones and transcription factors (6). TIP60 could therefore cooperate with ΔNp63α (or other transcription factors) at the ABCC1 regulatory region, modifying chromatin and/or acetylating ΔNp63α or co-factors to enhance transcription. TIP60 might also act through parallel routes, such as acetylation of NRF2 or NF-κB family members, which have been implicated in regulating ABCC family genes (59, 60, 61, 62). Together, these models highlight that TIP60 and ΔNp63α may function in a linear hierarchy or in a cooperative fashion to modulate ABCC1 expression. Although our data showing inhibition of ABCC1 with MK-571 mirrors data obtained with gene silencing of ABCC1, we acknowledge the potential off-target activity of MK-571. Use of additional or more selective inhibitors of ABCC1 would help confirm our findings. Further, additional chromatin-based studies (*e.g.*, ChIP analysis of TIP60 or ΔNp63α occupancy at the ABCC1 and XPC promoters) would further clarify this mechanism.

Cisplatin resistance is driven by enhanced DNA repair capacity and involves alterations in the transcriptional control and chromatin-based mechanisms in the regulation of nucleotide excision repair (NER), homologous recombination (HR), Fanconi Anemia (FA) and mis-match repair (MMR) pathways (1, 63). These DNA damage repair mechanisms play critical roles in the repair of cisplatin-induced DNA crosslinks. TIP60, a multifunctional histone acetyltransferase, modifies chromatin and acetylates non-histone proteins, including ΔNp63α and NER factors such as XPF, thereby influencing repair capacity and thus cisplatin resistance (11, 33). Therefore, the link between TIP60, p63, and XPC likely involves a combination of transcriptional control and chromatin-based mechanisms in the regulation NER, HR, FA and MMR pathways.

Prior studies have shown that TIP60 acetylates XPF to promote ERCC1 binding and NER activity (35, 64, 65). Our data showed that TIP60 may promote NER associated repair functions by positively regulating XPC and ERCC1 expression. Inhibition of the NER component XPB with spironolactone increased residual cisplatin-DNA adducts in resistant cells, supporting the functional relevance of TIP60 in the NER pathway. These findings are consistent with earlier studies showing both TIP60 and ΔNp63α are independently involved in NER and UV-induced DNA repair (33, 35, 64, 65). p63 has been implicated in the regulation of XPC levels and repair of UV-induced 6-4 photoproducts (6-4 PP) (33), yet the specific involvement of p63 isoforms in the regulation of XPC and the related NER pathway remains to be elucidated.

Interestingly, our data showed that both ΔNp63α and TIP60 also significantly increased the expression of the NER gene XPC, consistent with enhanced removal of cisplatin-DNA adducts in resistant cells and highlighting the need for additional mechanistic studies to determine if ΔNp63α and TIP60 are mediating these effects in a linear or parallel manner. TIP60 stabilizes ΔNp63α, which may drive XPC transcription, possibly with VDR involvement (11, 33). Alternatively, ΔNp63α could recruit TIP60 to XPC regulatory elements where its HAT activity would be expected to open chromatin and/or acetylates transcriptional regulators to enhance transcription. Finally, TIP60 may independently modify NER factors or other transcription factors to regulate XPC and overall, NER capacity (35). Although our current data do not distinguish between these models, they highlight promising avenues for future investigation.

ΔNp63 isoforms are well-known regulators of genes critical for epidermal homeostasis and DNA repair, and p63 depletion reduces XPC expression and impairs global genome NER, effects that may involve the VDR axis (33, 66). Our previous work showed that the Vitamin-D receptor (VDR) is overexpressed in non-melanoma skin cancer and is a known downstream target of ΔNp63α (67, 68). Recent studies have shown that VDR and p63 coordinate the release of XPC from DNA lesions, facilitating efficient NER (33). Investigating the TIP60/ΔNp63α/VDR axis will provide further insights into the broader regulation of NER and DNA repair in resistant cancers.

We further showed that TIP60 promotes cisplatin resistance by enhancing expression of the DDR genes ATM and RAD50, implicating TIP60 in the modulation of the HR pathway. TIP60 acetylates and activates ATM, triggering DNA damage signaling cascades, and regulates chromatin accessibility and transcription of key DNA repair genes such as BRCA1 another RAD family protein RAD51, thereby influencing homologous recombination efficiency (13, 25, 69, 70, 71). HR-deficient cancers tend to be more sensitive to crosslinking agents such as cisplatin (72, 73), and cancers with BRCA1 mutations have been observed to develop resistance to cisplatin (36). Importantly, TIP60 acetylates and stabilizes ΔNp63α, which transcriptionally upregulates RAD18, another crucial component of the HR pathways (29). RAD18 in turn activates PCNA through ubiquitination (74), further linking TIP60 and ΔNp63α to the trans-lesion synthesis repair pathway.

Our observation that TIP60 enhances FANCD2, a known transcriptional target or ΔNp63α also implicates the TIP60/ΔNp63α axis in FA repair. Mono-ubiquitination of FANCD2 is essential for repair of cisplatin-induced DNA damage (75). De-ubiquitination of FANCD2, which promotes its activation and DNA repair function, is facilitated by the ubiquitin-specific protease USP1 (76, 77). Notably, ΔNp63α upregulates USP1, which deubiquitinates and activates FANCD2, further integrating the axis into FA repair (29). In addition, TIP60 enhances FANCD2 expression and facilitates FANCD2 chromatin localization (13, 78). Together these findings suggest a complex, yet still unexplored, crosstalk between TIP60 and ΔNp63α across multiple DDR pathways that contribute to chemoresistance.

Because TIP60 is a central chromatin regulator and acetyltransferase with known roles across multiple DDR pathways (79), we cannot exclude that its effects on ABCC1 and XPC are partly parallel. TIP60 has been implicated in Fanconi anaemia-mediated interstrand crosslink repair (ICL) and homologous recombination (HR) and is required for efficient repair of ICLs and cisplatin resistance (13, 78), placing it upstream of a cascade of DDR responses that influence transcriptional regulation. Moreover, TIP60 directly acetylates NER component, XPF to activate repair complexes, and broadly regulates chromatin marks at DNA-damage response loci and such changes in DDR protein activity or chromatin state could secondarily alter transcription factors or transcriptional networks that control DNA repair (35). Additional studies are required to determine whether TIP60 and ΔNp63α influence the trans-lesion synthesis (TLS) and the Fanconi Anemia (FA) repair pathways in ways that further contribute to cisplatin resistance.

From a translational perspective, the combination of ionizing radiation (IR) and cisplatin remains a standard of care for many SCCs (80). Our findings suggest for the first time, that TIP60 inhibitors may sensitize cisplatin resistant tumors, particularly cutaneous SCC and HNSCC. Since TIP60 also facilitates repair of UV induced cyclobutane pyrimidine dimers (CPDs) and 6-4 photoproducts (6-4 PPs), and ionizing radiation induced DSBs (double strand breaks) and SSB (single strand breaks), targeting TIP60 could impair multiple DNA repair pathways. TIP60 inhibitors NU9056 and TH1834 have already demonstrated efficacy in animal model studies (35, 43, 44, 81, 82, 83) warranting further exploration of their combination with IR and cisplatin in patient-derived xenograft (PDX) models. Our use of the TIP60 acetyltransferase inhibitor TH1834 phenocopied the effects of TIP60 depletion, producing similar reductions in ABCC1 and XPC expression and corresponding changes in cisplatin-DNA adduct levels and cellular response to cisplatin. Future experiments using a catalytically deficient TIP60 mutant may provide this additional mechanistic insight. Finally, although our data supports the mechanistic contribution of TIP60, ABCC1-mediated efflux, and NER-dependent repair to the cisplatin response, a formal systematic dose–matrix combination analysis to formally quantify the combined effects of TH1834 with MK-571 or spironolactone performed using Bliss Independence and Loewe Additivity models is an important next step in determining the potential therapeutic potential of TIP60-inhibition strategies (84).

In conclusion, our study provides compelling evidence that TIP60 is a critical mediator of cisplatin resistance in SCC cells through its dual functions in regulating drug efflux *via* ABCC1 and promoting NER associated removal of cisplatin-DNA adducts through XPC. We demonstrate that TIP60 integrates complementary mechanisms that promote chemoresistance. TIP60 drives cell proliferation and survival under cytotoxic stress while also limiting cisplatin-induced DNA damage by facilitating removal of cisplatin-DNA adducts. Concurrently, TIP60 enhances drug efflux by upregulating transporters that reduce intracellular cisplatin accumulation. These coordinated effects establish TIP60 not only as a marker but as a pivotal driver of resistance, aligning DNA repair-associated pathways and efflux pathways to determine cellular sensitivity. Our data showing that pharmacological inhibition of TIP60 sensitizes resistant cells by increasing cisplatin-DNA adduct levels and impairing repair highlight TIP60 as a compelling therapeutic target for strategies aimed at overcoming cisplatin resistance across different cancers. Targeting the TIP60/ΔNp63α axis could be a novel strategy to overcome platinum resistance and improve outcomes for patients with platinum-resistant SCC.

## Experimental procedures

### Cell lines and reagents

The cisplatin-resistant A431 variant cell line (A431 Pt) and the corresponding Parental cell line (A431 Parental) were a generous gift from Dr Paola Perego (Istituto Tumori di Milano, Milan, Italy). Derivation of these lines was described previously (39). A431 Parental and A431 Pt cells were cultured for no more than 20 passages to avoid morphological changes associated with extended culture. The naturally resistant Head and Neck SCC JHU006 and sensitive JHU029 cell lines were obtained from Dr James W Rocco (Ohio State University). All cell lines were maintained in RPMI 1640 medium supplemented 10% FBS and 250 U penicillin and 250 μg streptomycin (85). The Human pharyngeal SCC cell line Detroit-562 was a generous gift from Dr Weiwen Long (Wright State University) and maintained in Eagle’s minimum essential media (EMEM) supplemented with 8% FBS and 250 U penicillin and 250 μg streptomycin. A431 and JHU029 cells stably expressing TIP60 or eGFP as a control were generated by lentiviral-mediated transduction of A431 and JHU029 cells and maintained in blasticidin antibiotic selection as described previously (11).

### Reagents, chemicals and Inhibitors

Cisplatin (cis-diammineplatinum (II) dichloride) and the XPB inhibitor Spironolactone were purchased from Sigma-Aldrich. A 1 mg/ml cisplatin stock was prepared in 1X PBS. The TIP60 specific inhibitor TH1834 and the ABCC1 inhibitor MK-571 were purchased from MedChemExpress (Monmouth Junction, NJ, USA).

### Cisplatin treatments

Cisplatin treatments were conducted as 2-h pulse exposure followed by 1X PBS wash and return to cisplatin free culture medium. For experiments assessing removal of cisplatin-DNA adducts, the 2-h post-pulse time point was defined as “time 0,” and genomic DNA was isolated at the indicated time points thereafter. Adduct levels were quantified relative to the time 0 baseline to evaluate the removal of cisplatin-DNA lesions under drug-free conditions.

This exposure paradigm was selected based on prior characterization of these cell models and our previous studies, allowing consistency with established experimental conditions. Pulse treatments were based on our previously reported IC50 values for A431 Parental (12.5 μg/ml, 42 μM), A431 Pt (42 μg/ml, 140 μM), JHU029 (4.5 μg/ml, 15 μM), JHU026 (9 μg/ml, 30 μM), and Lenti-A431 (14 μg/ml, 47 μM) (10). Because these experiments use a short pulse-treatment paradigm to model transient drug exposure, the concentrations used are higher than those typically used in continuous treatment models (86).

### Quantitation of DNA-bound cisplatin by ICP-mass spectrometry

DNA-bound cisplatin was quantitated in A431 Parental and Pt cells pulsed for 2 h with either vehicle (PBS) or 42 μg/ml (140 μM) cisplatin, washed with 1X PBS, and subsequently incubated with vehicle (DMSO) or 50 μM TH1834 for 24 h. Genomic DNA was GenElute Mammalian Genomic DNA miniprep kit from Sigma-Aldrich. DNA (10–30 μg) was hydrolyzed overnight in 1% nitric acid at 70 °C in 200 μl. Samples were then analyzed by ICP-mass spectrometry at Keystone Bioanalytical (North Wales, PA).

### Measurement of Intracellular Drug Accumulation by ICP-MS

A431 Parental and Pt cells were seeded in 6-well plates, pulsed for 2 h with 42 ug/ml (140 μM) cisplatin, washed with 1X PBS and incubated in cisplatin-free media for an additional 2 h. Sufficient replicates were included to allow sampling at 0, 15, 30, 60, 120 and 240 min from the start of the experiment (*i.e.* throughout the pulse and washout periods). Cells were then lysed as reported previously (50). Briefly, concentrated (50–70%) nitric acid (1 ml) was added to each well, and the plate was incubated overnight at room temperature. The following day, samples were moved into Omni-vials and incubated at 65 °C overnight to thoroughly dissolve all cellular debris. The following day, the nitric acid was diluted with 4 ml of 0.1% Triton X-100 in ddH_2_O and incubated again at 65 °C overnight. The absolute concentrations of elemental platinum were determined by inductively coupled plasma mass spectrometry (ICP-MS) using a PerkinElmer Element two inductively coupled plasma mass spectrometer (PerkinElmer Life and Analytical Sciences). Analysis was performed by Keystone Bioanalytical, a commercial vendor with established SOPs for this analysis. Platinum quantitation by ICP-MS uses certified external calibration standards prepared with known concentrations of platinum to generate a standard curve; all sample values in our study fell within the validated linear range of the curve, as confirmed by the vendor's quality control metrics.

### Transient transfections, siRNA and plasmids

TIP60, XPC and ABCC1 silencing was performed by one round of transient siRNA transfection using Lipofectamine RNA-iMax per manufacturer’s instructions (Life Technologies) with cells harvested 48 h post-transfection as described previously (66). Allstars non-silencing control (NSC), p63, and TIP60 siRNA were purchased from Qiagen. siRNA for the 3′UTR of TIP60 designed to knock down endogenous TIP60 while leaving exogenous TIP60 intact was ordered from Dharmacon (Lafayette, CO, USA). The target sequences used for siRNA were as follows: NSC siRNA (5′-TTGGATGTTAGTAACAACAAT-3′), p63 siRNA_1 (5′-CACCCTTATAGTCTAAGACTA-3′), siTIP60 (5′-CACCACATTGCCTGTCCTCTA-3′), siTIP60-3′UTR (5′-GTACAGAGGGCTGGTGATTTT-3′ and 5′-GGTGGGTGCTGGCTGCAAATT-3′), siABCC1 (5′-CTCAATGGGATCAAAGTGCTA-3′) and siXPC (5′-TCGGAGGGCGATGAAACGTTT-3′). ΔNp63α is the predominant p63 isoform expressed in HNSCC tumors (87) and in cell lines used in this study as evidenced by prior reports (88) and our immunoblot data. Accordingly, sip63 primarily targets ΔNp63α in this experimental system and all relevant text and figures refer to ΔNp63α. Transient transfections were performed using 40 nM of siRNA against the target gene or 40 nM (NSC) siRNA, as performed previously (11).

### Immuno-dot blot assay for quantitation of intrastrand 1,2-d(GpG) cisplatin-DNA adducts

Cisplatin treated cells were washed in PBS, harvested using trypsin, and washed with ice-cold PBS. Total genomic DNA was purified using GenElute Mammalian Genomic DNA miniprep kit from Sigma-Aldrich. DNA concentrations were measured using NanoDrop spectrophotometer (Thermo Fisher Scientific Inc). DNA samples were diluted and heat-denatured for 10 min at 95 to 100 C and neutralized with cold 2M ammonium acetate pH 7.0 (Fisher Scientific). Equal amounts of genomic DNA (100–200 μg) were spotted onto a nitrocellulose membrane in duplicate using a 96-well dot-blot apparatus, as described previously (40). Blots were blocked in milk and probed with rat anti-1,2-d(GpG)-cisplatin-DNA adduct antibody [CP9/19] (ab103261) (Abcam) and HRP-coupled anti-rat IgG secondary antibody (Abcam). Chemiluminescence was visualized with Clarity Western ECL substrate (Bio-Rad) or SuperSignal West Femto substrate (Thermo Scientific) using a Molecular Imager Chemi-Doc XRS + imaging system (Bio-Rad). Image Lab (Bio-Rad) was used to quantify signal intensities. Blots were stained with SYBR Gold (Invitrogen) to confirm equivalent DNA loading. Quantitation of dot blot assays was performed within the linear detection range for both DNA input and antibody signal, consistent with established protocols (89).

### Quantitative RT-PCR

Total-RNA isolation from human cell lines was performed using the E.Z.N.A. Total RNA kit according to the manufacturer protocol (Omega Bio-Tek). cDNA was synthesized from 1 μg of total RNA using the qScript cDNA SuperMix (Quantabio). qRT-PCR was performed using the Applied Biosystem 7900HT or 7 Flex Real-Time PCR systems using Assays on Demand (AOD). A DDR screen was performed using mRNA targets selected from the Qiagen DNA Damage Signaling Pathway RT2 Profiler PCR array (GeneGlove ID: PAHS-029Z) and ThermoFisher Human DNA Damage Signaling Panels. Life Technologies assay IDs for all DDR genes tested are provided in Table S1. Additionally, AODs specific for human GAPDH (4325792), β-globulin (Hs00187842_m1), ATM (Hs01112355_g1), CHEK2 (Hs00200485_m1), ERCC1 (Hs01012158_m1), FANCD2 (Hs00276992_m1), GADD45A (Hs00169255_m1), RAD50 (Hs00990023_m1), and XPC (Hs01104206_m1), ABCC1 (Hs01561483_m1) (Thermo Fisher, Waltham, MA, USA) were used with each sample run in technical triplicate. Relative expression was calculated using the ΔΔCT method with GAPDH used n as an endogenous control as described previously (90, 91).

### Immunoblot analysis

Cells were harvested using trypsin, washed in PBS, and lysed in ice-cold phosphatase inhibitor buffer (50 mM Tris-HCl pH 8, 120 mM NaCl, 5 mM sodium pyrophosphate, 10 mM NaF, 30 mM para-nitrophenylphosphate, 1 mM benzamidine, 0.1% NP-40, 1% Triton X-100 and 0.2 mM PMSF, 100 nM sodium orthovanadate) supplemented with 10% protease inhibitor cocktail (Sigma) to 1% final concentration (Sigma). Lysed cells were incubated on ice for 30 min with a gentle mixing every 10 min and cell lysates were clarified using centrifugation for 5 min at 14,000*g* at 4 C. Total protein concentrations were determined by BCA assay (Thermo Fisher Scientific Inc). Equivalent amounts of protein were resolved on either 10% or 4 to 20% gradient SDS-polyacrylamide gels as appropriate for the MW of the proteins being resolved and transferred to polyvinylidene difluoride (PVDF) membranes. Proteins were detected using anti-p63 (4A4) mouse monoclonal (for detection of the dominant p63 isoform ΔNp63α expressed in the cell lines used in this study), mouse anti-TIP60 (C7, Santa Cruz Biotechnology, Santa Cruz, CA, USA), rabbit anti-ABCC1 (D5C1X) and PARP (9542) (Cell Signaling Technology, Danvers, MA, USA) and mouse XPC (D1M5Y), XPB (G10) and anti-β-actin monoclonal (Santa Cruz Biotechnology). Appropriate anti-rabbit or anti-mouse horseradish peroxidase-conjugated secondary antibodies (Promega, Madison, WI, USA) were used to facilitate chemiluminescence detection with Western Lightning Plus chemiluminescent kit (PerkinElmer) or SuperSignal West Femto Maximum Sensitivity Substrate (Thermo Fisher Scientific Inc). Blots were imaged on a Fujifilm LAS-3000 (Fujifilm Corp). Fold change in protein expression was calculated by normalizing band intensity to β-actin as a loading control.

### Cellular thermal shift assay (CETSA)

CETSA was performed as previously described with modifications (47). Cells were lysed in 1X PBS supplemented with Protease Inhibitor Cocktail (Sigma-Aldrich, St Louis, MO) by three freeze-thaw cycles in liquid nitrogen. Cell debris was removed by centrifugation at 17,000*g* for 20 min at 4 °C. Clarified whole-cell protein lysates were then treated with either vehicle (DMSO) or TH1834 (50 μM for Lenti-A431 TIP60 cells or 100 μM for JHU006 cells) for 30 min at room temperature with gentle mixing. Treated lysates were then aliquoted into PCR tubes (20 μl per tube) and subjected to a 3-min heat pulse at temperatures ranging from 38 °C – 54 °C using a temperature gradient VeriFlex thermocycler (Applied Biosystems). Following heating, samples were centrifuged at 17,000*g* for 20 min at 4 °C, and the soluble fraction was analyzed by standard immunoblot analysis using antibodies to TIP60 and β-actin. Band intensity was determined by densitometry. Melting temperature (T_m_) was determined by curve fitting in GraphPad Prism (v11.0) using a Boltzmann sigmoidal regression for CETSA as previously described (92).

### Anchorage dependent (2D) survival assay

Survival was assessed by a clonogenic colony forming assay as described previously (80, 93). Briefly, transfected or untreated cells were harvested with trypsin, suspended in RPMI-1640 1X complete media, counted and plated at 10,000 cells/well densities in triplicate. Plates were incubated in a 37° C humidified cell culture incubator at 5% CO_2_ for 24 h, pre-treated with vehicle, MK-571 or SP for 24 h, pulsed with the IC50 dose of cisplatin for 2 h, washed in PBS and returned to cisplatin-free medium containing vehicle, MK-571 or SP for an additional 7 to 14 days. Following aspiration and removal of cell culture media and dead, floating cells and a wash with 1X phosphate-buffered saline (PBS), cell colonies were stained and fixed with a 6% glutaraldehyde and 0.5% crystal violet solution in PBS. The crystal violet stain was then solubilized in 10% acetic acid and the absorbance at 590 nm measured. The absorbance values of all treatment samples were normalized to the control samples (set to 1.0) for each experiment.

### Soft agar (3D) colony formation assay

Anchorage-independent colony formation assays were performed as described previously using cells grown in 0.3% Ultrapure Agar purchased from Thermo Scientific in a 37° C humidified cell culture incubator at 5% CO_2_ (94). A431 Pt cells were transfected with NSC or siABCC1 or siXPC, pulsed with half the IC50 dose of cisplatin (6.25 μg/ml, 21 μM), washed with PBS, and fresh complete medium containing appropriate treatment (*e.g.*, MK-571, TH1834 or DMSO vehicle) was added to replace old media every 2 to 3 days for a total of 28 to 30 days. Cells were then stained overnight at 37° C in 5% CO_2_ with 1 mg/ml of Nitro blue tetrazolium chloride (NBT) (Electron Microscopy Sciences). Surviving colonies were imaged at 2X magnification on an Evos Cell Imaging System (Thermo Fisher Scientific Inc) and counted using ImageJ software.

### Statistical Analysis

Linear-mixed effects models implemented in R version 4.50 (nlme package, lme function) were used in studies with technical replication, with residual variance allowed to vary between groups and a random intercept term included to account for the correlation induced by technical replication. Due to non-linearity and heteroscedasticity (*i.e.*, variance increasing with dose), each dose-response curve was modeled as a polynomial (quadratic or cubic) and the variance was allowed to depend on dose. In studies without technical replication, two-sample t-tests allowing unequal variances were used to compare groups. In studies with more than two groups, a *t* test was used for each pairwise comparison to allow for unequal variances. In comparisons of relative levels in which one group was the reference and so fixed at a value of 1, a one-sample *t* test was used to compare to that fixed value. *p*-values ≤ 0.05 were considered statistically significant. *p*-values > 0.05 are indicated as nonsignificant (“ns”) in figures, as appropriate. Where statistical significance was not reached (*p* > 0.05), observed trends were consistent across multiple independent experiments and cell lines, suggesting reproducible biological effects even if the conventional significance threshold was not met.

## Data availability

All data are contained within the manuscript and the associated supporting information file.

## Supporting information

This article contains supporting information (91).

## Conflict of interest

The authors declare that they have no conflicts of interest with the contents of this article.
