# Supplementary material for: TIP60 promotes chemoresistance by limiting intracellular platinum accumulation and enhancing removal of cisplatin-DNA adducts
Source: J Biol Chem. 2026 Jul 16;302(9):113338. doi: 10.1016/j.jbc.2026.113338 (PMC13488049; doi:10.1016/j.jbc.2026.113338)
Supplement: Supplementary file 1 [file mmc1.pdf]

Supporting Information

TIP60 promotes chemoresistance by limiting intracellular platinum accumulation and enhancing removal of cisplatin-DNA adducts

Akshay Hira[1], Michael P. Craig[1], Caroline McLaughlin [1], Jin Zhang[1], John Turchi[2], Michael G. Kemp[3], Ramzi W. Nahhas[4,5], and Madhavi P. Kadakia[1,*]

Data included:

Figure S1: TIP60 inhibition increases DNA-bound cisplatin in resistant cells with low intracellular drug levels.

Figure S2: TIP60 knockdown decreases ABCC1 and XPC protein levels in Detroit-562 cells.

Figure S3: TH1834 engages both overexpressed and endogenous TIP60.

Figure S4: Knockdown of ABCC1 increases 1,2-d(GpG) cisplatin-DNA adduct levels

Figure S5: Cisplatin resistant cells exhibit enhanced removal of 1,2-d(GpG) cisplatin-DNA adducts.

Figure S6: Pharmacological inhibition of TIP60 impairs removal of 1,2-d(GpG) cisplatin-DNA adducts in resistant cells.

Figure S7: Knockdown of XPC increases 1,2-d(GpG) cisplatin-DNA adduct levels.

Table S1: DDR gene expression in A431 PT cells relative to Parental controls

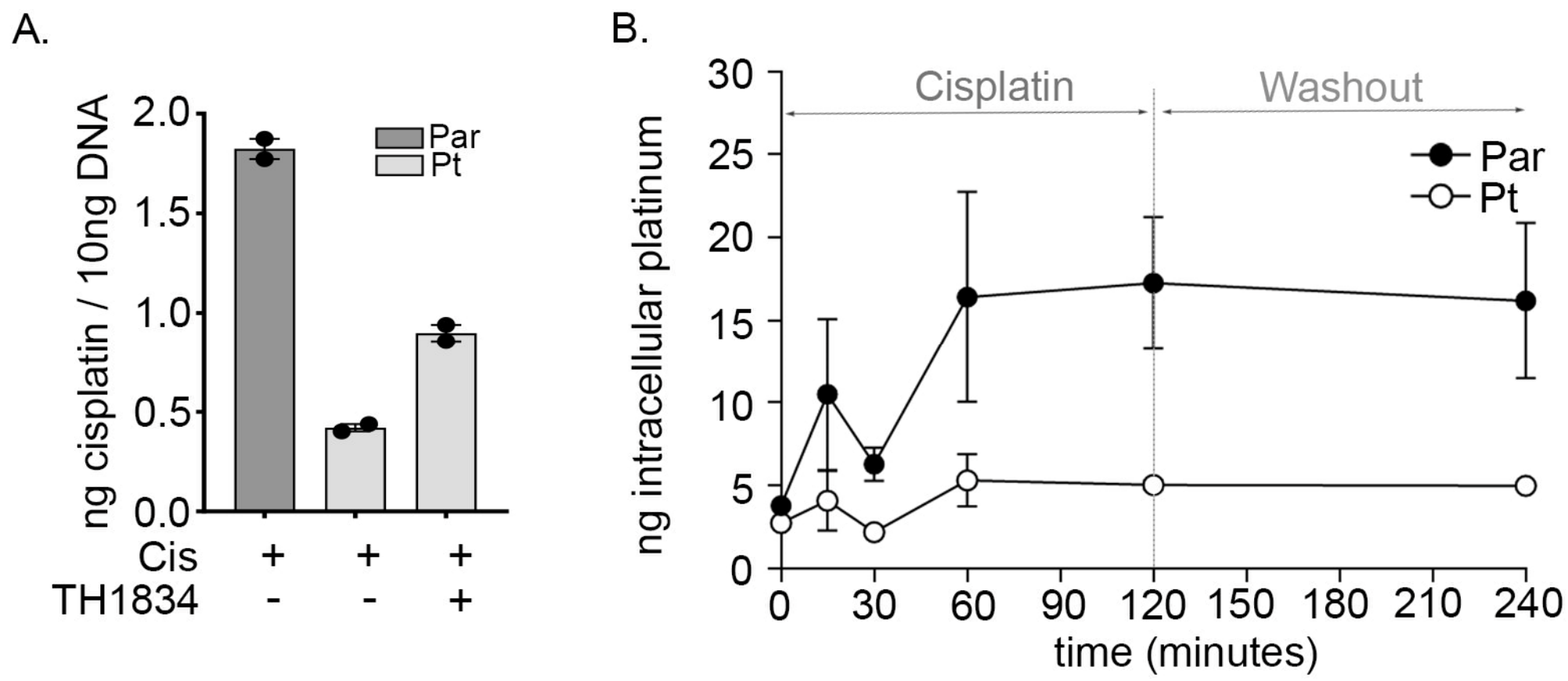

Figure S1: TIP60 inhibition increases DNA-bound cisplatin in resistant cells with low intracellular drug levels. (A) A431 Par and Pt cells were pulsed for 2 h with 42 µg/ml (42 µM) cisplatin, washed in 1X PBS and then incubated for 24 h in vehicle or 50 µM TH1834, a TIP60 inhibitor. At 24 h post-pulse, genomic DNA was isolated and hydrolyzed using nitric acid hydrolysis. DNA-bound cisplatin was quantitated by ICP-MS. Measurements are from n = 2 technical replicates. Error bars indicate +1 S.D. **(B)** Intracellular platinum levels were measured by ICP-MS throughout a 2 h cisplatin pulse with 42 µg/ ml cisplatin and subsequent 2 h washout/efflux in drug-free medium. Replicate plates of A431 Par and Pt were harvested at defined timepoints throughout the drug treatment and washout intervals. Cells were lysed and hydrolyzed in nitric acid as previously described (50). All data presented are the means of two independent experiments. Error bars indicate ± S.E.M. Connecting lines link individual data points (no regression applied) since the timeline comprises two distinct phases: cisplatin treatment and washout.

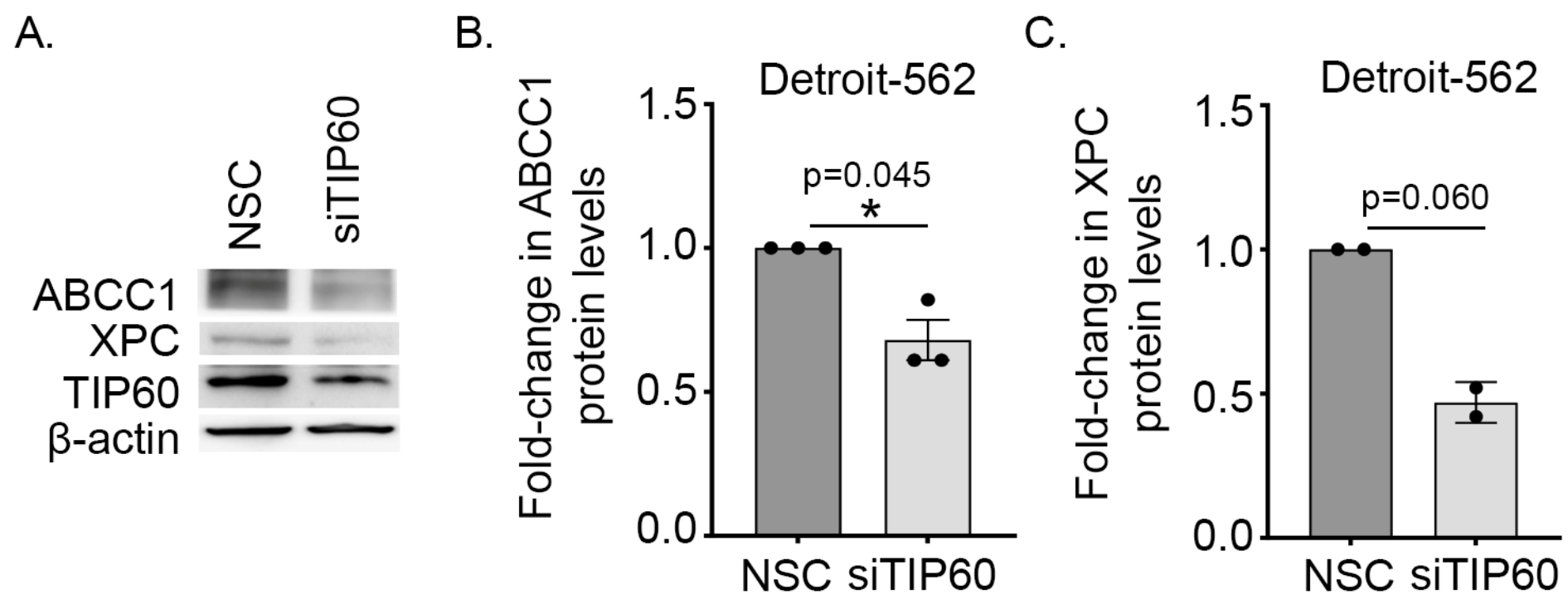

Figure S2: TIP60 knockdown decreases ABCC1 and XPC protein levels in Detroit-562 cells. Cisplatin-resistant Detroit-562 cells were transiently transfected with non-silencing control (NSC) or siTIP60 and harvested 48h later for immunoblot analysis of ABCC1 or XPC protein levels. (A) Representative immunoblots. Densitometric analysis of (B) ABCC1 and (C) XPC shows the fold change in protein levels relative to NSC controls. Error bars indicate +1 S.E.M from 3 (ABCC1) or 2 (XPC) independent biological replicates; *$p \leq 0.05$ calculated using one-sample t-tests.

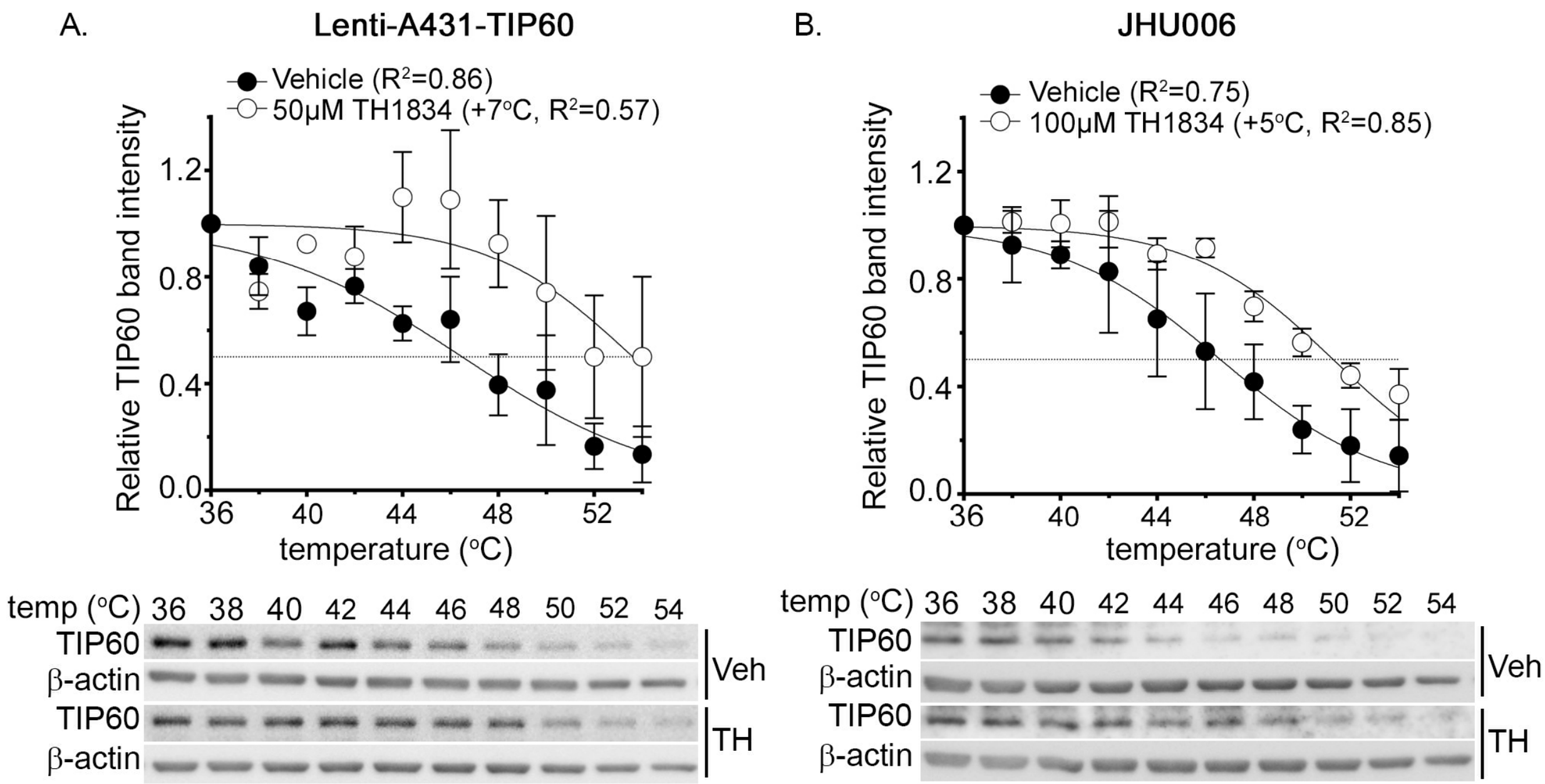

Figure S3: TH1834 engages both overexpressed and endogenous TIP60. Cellular thermal shift assays (CETSA) were performed on clarified cell lysates from **(A)** Lenti-A431 cells stable expressing TIP60, treated with 50 µM TH1834 and **(B)** JHU006 cells treated with 100 µM TH1834. Lysates were incubated with vehicle (DMSO) or TH1834 for 30 minutes at room temperature, aliquoted and subjected to a 3-minute heat pulse at the indicated temperatures (*x*-axis) using a temperature gradient thermocycler. The soluble fraction was analyzed by immunoblot (bottom panels) using antibodies specific TIP60 or β-actin. β-actin was included as a specificity control for ligand induced thermal stabilization. TIP60 and β-actin band intensities were quantified by densitometry. Y-axis indicates the fold change in TIP60 levels at each temperature relative to 36℃. R^2 values indicate the goodness of fit for the Boltzmann sigmoidal regression lines used to obtain T_m values. Error bars indicate +1 S.E.M from n = 2 (lenti-A431 TIP60) or n = 3 (JHU006) independent biological replicates. All runs showed positive thermal shifts with TH1834 treatment (relative to vehicle).

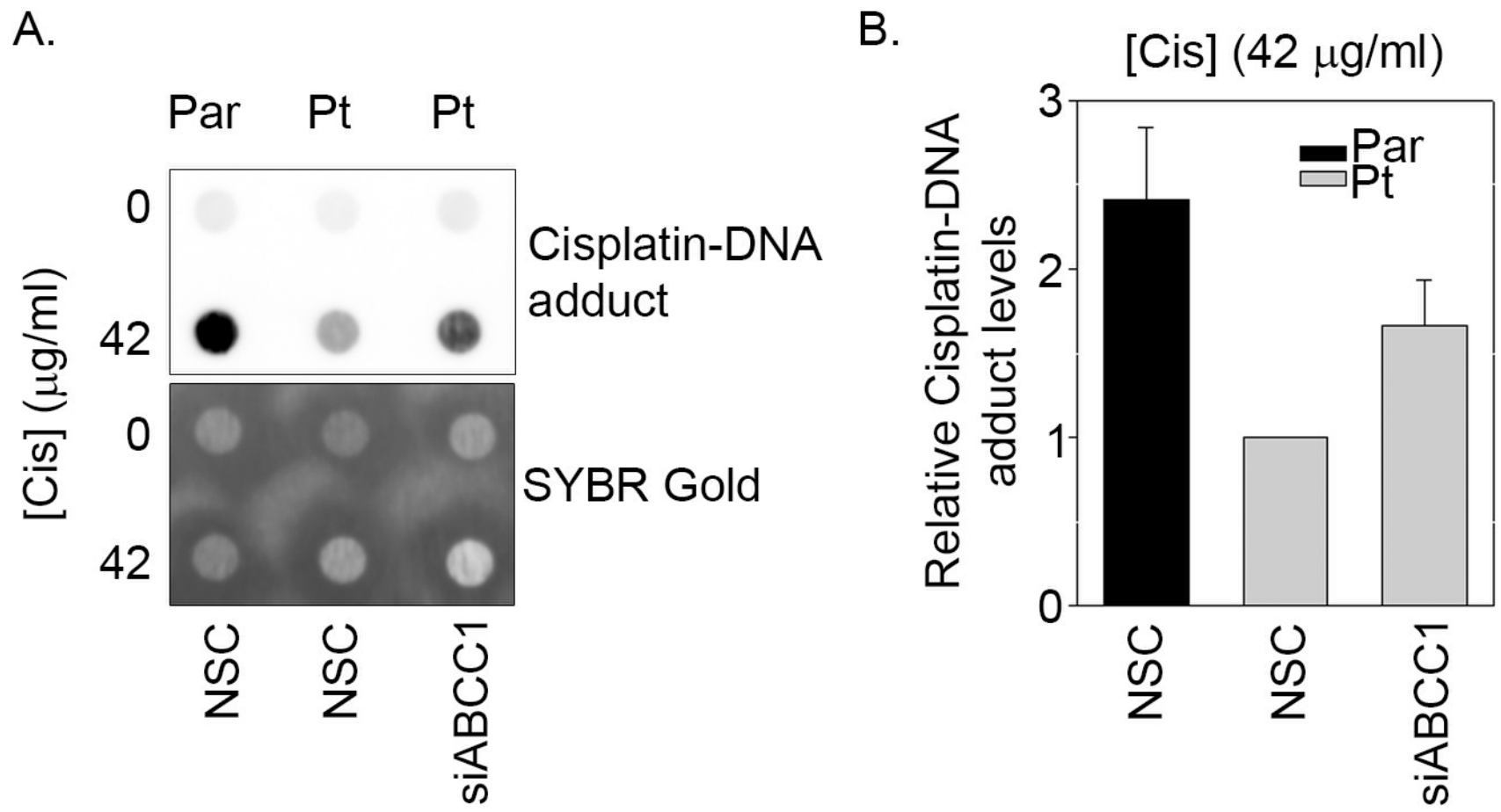

Figure S4: Knockdown of ABCC1 increases 1,2-d(GpG) cisplatin-DNA adduct levels. (A) A431 Pt cells were transfected with non-silencing control (NSC) or si-RNA against ABCC1, treated with vehicle or pulsed with 42 µg/ml cisplatin for 2 hours and harvested at 0h. Sensitive cells transfected with non-silencing control (NSC) served as a control. Immuno-dot blots of genomic DNA were probed with an antibody that specifically recognizes 1,2 d(GpG) cisplatin DNA adducts and SYBR Gold staining was used to confirm equal DNA loading. (B) Densitometric quantitation of adduct levels in ABCC1 knockdown cells relative to A431 Pt NSC at 42 µg/ml dose of cisplatin. The measured signal represents cisplatin-DNA adduct levels following pulse exposure, reflecting combined effects of uptake, efflux, and DNA reactivity during the treatment window. Error bars indicate +1 S.E.M from 3 independent biological replicates; p-value calculated using one-sample t-tests between the groups being compared. "ns" indicated non-significance ($p > 0.05$).

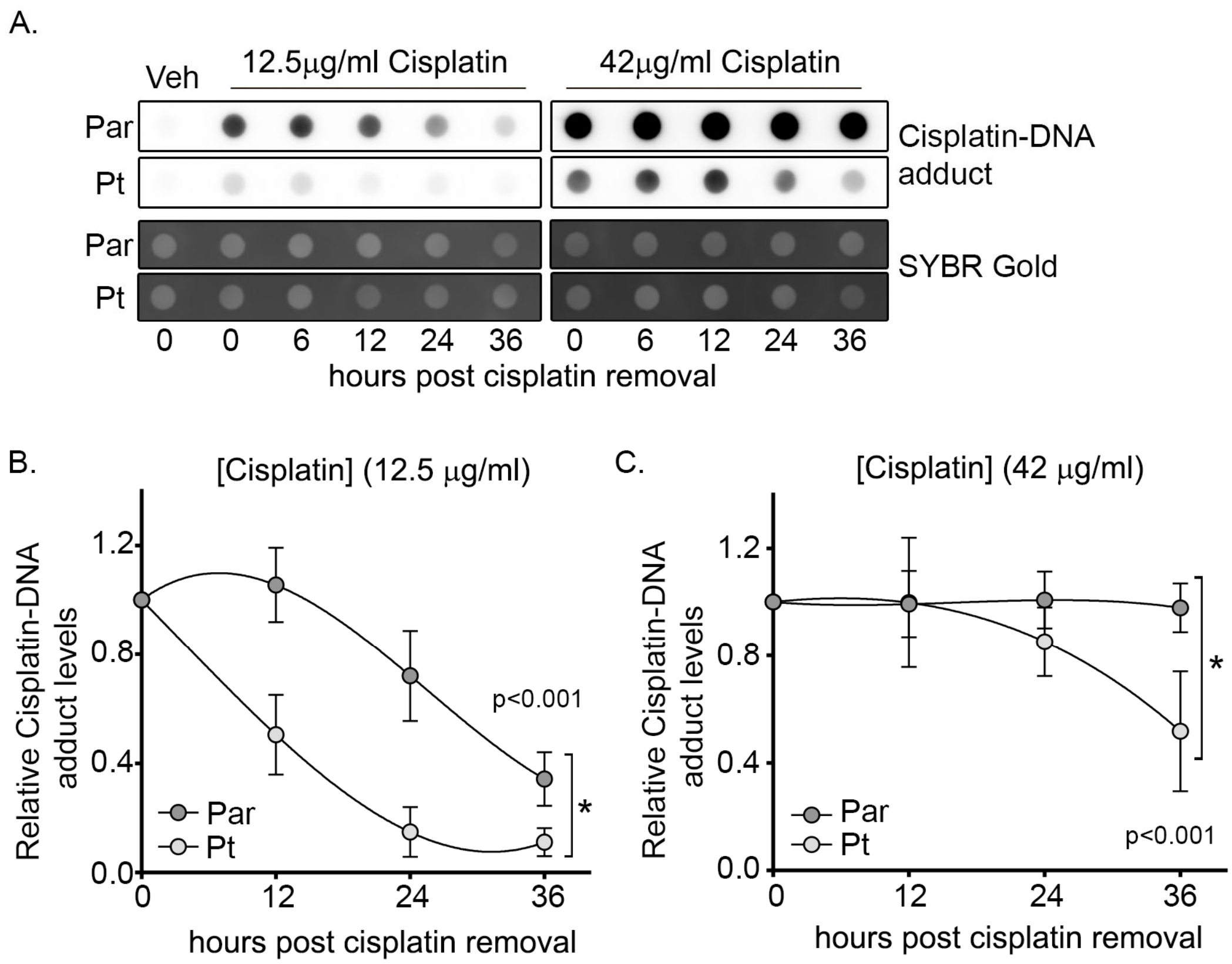

Figure S5: Cisplatin resistant cells exhibit enhanced removal of 1,2-d(GpG) cisplatin-DNA adducts. (A) A431 Parental and Pt cells were treated with either 12.5 µg/ml or 42 µg/ml for 2 hours. Following treatment cells were washed with PBS and incubated in cisplatin-free medium for remainder of the experiment. The 2-hour post-treatment time point was defined as time 0h and genomic DNA was isolated at the indicated time points (0, 12, 24 and 36 hours) to measure remaining cisplatin adduct levels. Immuno-dot blots of genomic DNA were probed with an antibody that specifically recognizes 1,2 d(GpG) cisplatin DNA adducts and SYBR Gold staining was used to confirm equal DNA loading. (B, C) Densitometric quantitation with Y-axis showing cisplatin-DNA adduct levels relative to the post-pulse baseline (time 0) to evaluate removal of cisplatin-DNA adducts following pulse exposure. The measured signal represents cisplatin-DNA adduct levels following pulse exposure, reflecting combined effects of uptake, efflux, and DNA reactivity during the treatment window. Regression lines shown are third order polynomial (cubic) lines of best fit. Error bars represent ±1 S.E.M from 3 independent biological replicates. Statistical significance was determined using a mixed-effects model with time as a fixed effect (*$p \leq 0.05$).

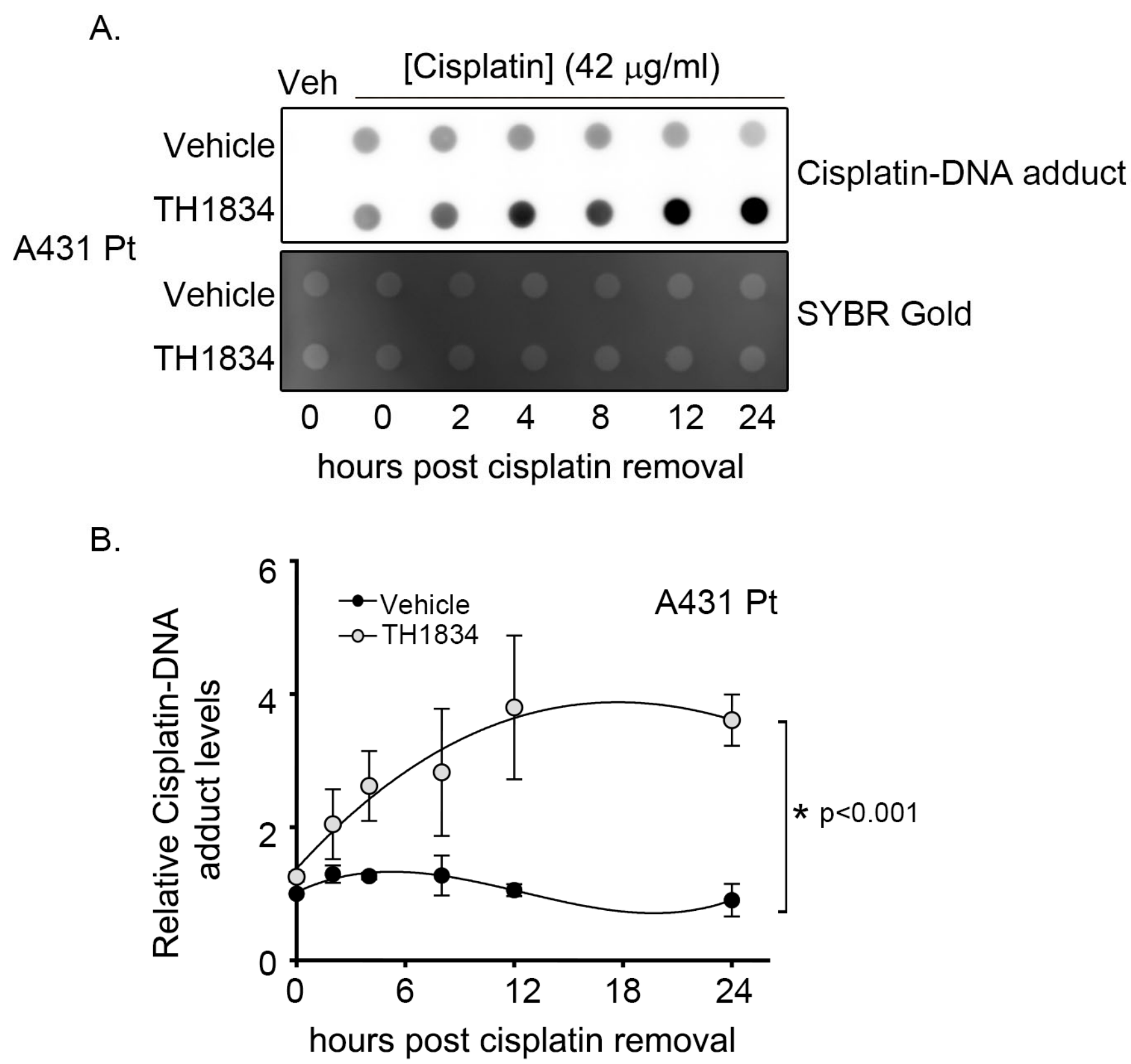

Figure S6: Pharmacological inhibition of TIP60 impairs removal of 1,2-d(GpG) cisplatin-DNA adducts in resistant cells. A431 Pt cells were treated with 42 µg/ml cisplatin for 2 hours, followed by PBS wash and incubation in cisplatin free complete medium. The post-pulse time point was defined as time 0. Cells were subsequently treated with either vehicle (DMSO) or 50 µM TH1834 and harvested at the indicated timepoints. Immuno-dot blots of genomic DNA were probed with an antibody that specifically recognizes 1,2 d(GpG) cisplatin DNA adducts and SYBR Gold staining was used to confirm equal DNA loading. **(B)** Densitometric quantitation with Y-axis showing cisplatin-DNA adduct levels relative to the post-pulse baseline (time 0). The measured signal represents cisplatin-DNA adduct levels following pulse exposure, reflecting combined effects of uptake, efflux, and DNA reactivity during the treatment window. Regression lines shown are third order polynomial (cubic) lines of best fit. Error bars represent ±1 S.E.M from 2 independent biological replicates. Statistical significance was determined using a mixed-effects model comparing treatment groups over time ($^*p \leq 0.05$).

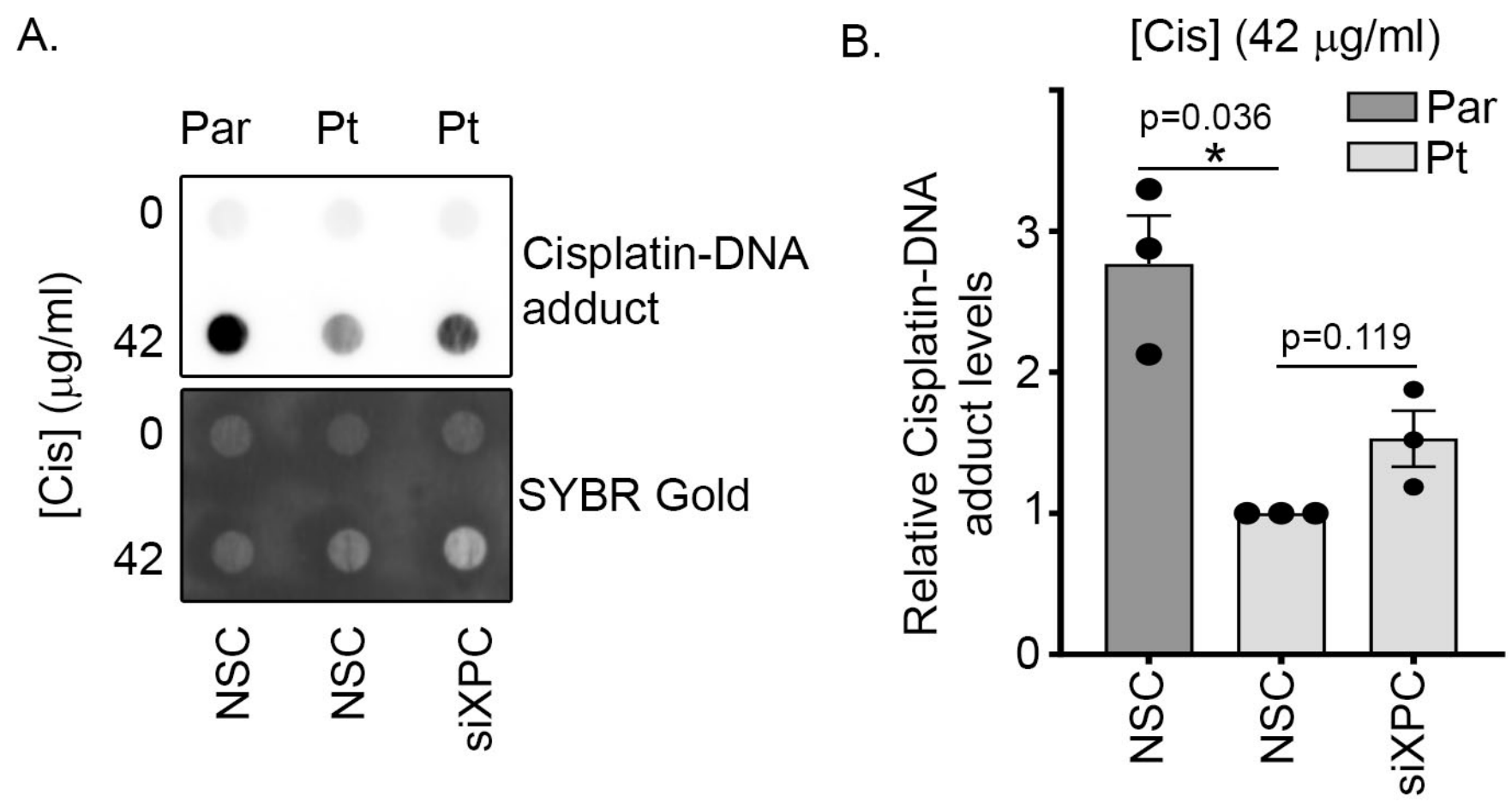

Figure S7: Knockdown of XPC increases 1,2-d(GpG) cisplatin-DNA adduct levels. (A) A431 Pt cells were transfected with non-silencing control (NSC) or si-RNA against XPC, followed by treatment with the indicated cisplatin dose for 2 hours. Cells were than washed with PBS and harvested immediately for genomic DNA isolation. Cisplatin sensitive A431 parental cells transfected with non-silencing control (NSC) served as a control. Immuno-dot blots of genomic DNA were probed with an antibody that specifically recognizes 1,2 d(GpG) cisplatin DNA adducts and SYBR Gold staining was used to confirm equal DNA loading. (B) Densitometric quantitation of the net cisplatin-DNA adduct burden levels following pulse exposure. The measured signal represents cisplatin-DNA adduct levels following pulse exposure, reflecting combined effects of uptake, efflux, and DNA reactivity during the treatment window. The y-axis represents adduct levels in XPC knockdown cells relative to A431 Pt control cells treated with 42 μg/ml dose of cisplatin. Error bars represent +1 S.E.M from 3 independent biological replicates. Statistical significance was determined using one-sample t-tests, comparing each condition to the A431 Pt control group (*$p \leq 0.05$). “ns” indicated non-significance ($p > 0.05$).

Table S1: DDR gene expression in A431 PT cells relative to Parental controls. Numbers listed indicate fold-change ± 1 standard deviation (S.D.) in transcript levels by RT-PCR. Each sample was run in technical triplicate. Relative expression was calculated using the ΔΔCT method with GAPDH used as an endogenous control as described in Livak et al 2001 (90) and Pfaffl et al 2001 (91).

Gene name	Fold change ± 1 S.D. in A431 Pt relative to Par (control)	AOD (Thermo Fisher)
ABL1	0.62 ± 0.06	Hs01104728_m1
APEX1	2.32 ± 0.16	Hs00172396_m1
ATM	1.43 ± 0.09	Hs01112355_g1
BARD1	1.03 ± 0.05	Hs00184427_m1
BAX	1.21 ± 0.08	Hs00180269_m1
BBC3	1.82 ± 0.18	Hs00248075_m1
BLM	1.10 ± 0.07	Hs00172060_m1
BRCA1	1.35 ± 0.12	Hs01556193_m1
BRIP1 (FANCJ)	0.91 ± 0.06	Hs00908143_m1
CDC25A	0.71 ± 0.03	Hs00947994_m1
CDC25C	1.03 ± 0.04	Hs00156411_m1
CDKN1A	2.82 ± 0.15	Hs00355782_m1
CHEK1	1.25 ± 0.06	Hs00967506_m1
CHEK2	2.90 ± 0.16	Hs00200485_m1
CSNK2A2	2.12 ± 0.18	Hs00176505_m1
DDB1	1.25 ± 0.03	Hs00172410_m1
DDB2	0.89 ± 0.07	Hs03044953_m1
ERCC1 (XPF)	1.17 ± 0.03	Hs01012158_m1
ERCC2 (XPD)	1.16 ± 0.10	Hs00361161_m1
EXO1	0.85 ± 0.05	Hs01116190_m1
FANCA	0.84 ± 0.07	Hs01116668_m1
FANCD2	1.10 ± 0.18	Hs00276992_m1
FANCG	0.69 ± 0.05	Hs00184947_m1
FEN1	0.95 ± 0.04	Hs00748727_s1
GADD45A	1.40 ± 0.05	Hs00169255_m1
MBD4	1.69 ± 0.10	Hs00187498_m1
MLH1	1.20 ± 0.07	Hs00179866_m1
MLH3	2.22 ± 0.12	Hs00998142_m1
MPG	1.98 ± 0.12	Hs01012594_m1
MSH2	0.61 ± 0.04	Hs00953523_m1
MSH3	1.30 ± 0.04	Hs00989003_m1
NBN	0.93 ± 0.07	Hs00159537_m1
NTHL1	1.30 ± 0.08	Hs00267385_m1
OGG1	0.99 ± 0.01	Hs00213454_m1

P73	1.45 ± 0.15	Hs01056230_m1
PARP1	1.35 ± 0.15	Hs00242302_m1
PMS1	1.34 ± 0.08	Hs00922262_m1
PMS2	0.87 ± 0.06	Hs00241053_m1
PNKP	1.31 ± 0.11	Hs00892544_m1
PPP1R15A	1.17 ± 0.09	Hs00169585_m1
PPM1D	0.86 ± 0.05	Hs01013292_m1
PRKDC	0.84 ± 0.03	Hs00179161_m1
RAD18	2.02 ± 0.08	Hs00892551_m1
RAD21	1.57 ± 0.07	Hs00366726_m1
RAD50	1.27 ± 0.08	Hs00990023_m1
RAD51	1.68 ± 0.05	Hs00947967_m1
RAD51B	2.06 ± 0.10	Hs01568763_m1
REV1	1.24 ± 0.04	Hs01019768_m1
RNF8	1.62 ± 0.06	Hs00187634_m1
RPA1	1.97 ± 0.07	Hs00161419_m1
SIRT1	0.92 ± 0.09	Hs01009006_m1
SMC1A	1.76 ± 0.07	Hs00196849_m1
SUMO1	0.83 ± 0.08	Hs02339312_g1
UNG	0.87 ± 0.05	Hs01037093_m1
XPA	0.96 ± 0.06	Hs00166045_m1
XPC	1.26 ± 0.14	Hs01104206_m1
XRCC1	1.87 ± 0.11	Hs00959834_m1
XRCC2	2.14 ± 0.25	Hs03044154_m1
XRCC3	1.90 ± 0.16	Hs00193725_m1
XRCC6	0.66 ± 0.04	Hs00995282_g1
